# The Serine Protease EspC from Enteropathogenic *Escherichia coli* Regulates Pore Formation and Cytotoxicity Mediated by the Type III Secretion System

**DOI:** 10.1371/journal.ppat.1005013

**Published:** 2015-07-01

**Authors:** Julie Guignot, Audrey Segura, Guy Tran Van Nhieu

**Affiliations:** 1 Equipe Communication Intercellulaire et Infections Microbiennes, Centre de Recherche Interdisciplinaire en Biologie (CIRB), Collège de France, Paris, France; 2 Institut National de la Santé et de la Recherche Médicale U1050, Paris, France; 3 Centre National de la Recherche Scientifique UMR7241, Paris, France; 4 MEMOLIFE Laboratory of Excellence and Paris Science Lettre, Paris, France; Osaka University, JAPAN

## Abstract

Type III secretion systems (T3SSs) are specialized macromolecular machines critical for bacterial virulence, and allowing the injection of bacterial effectors into host cells. The T3SS-dependent injection process requires the prior insertion of a protein complex, the translocon, into host cell membranes consisting of two-T3SS hydrophobic proteins, associated with pore-forming activity. In all described T3SS to date, a hydrophilic protein connects one hydrophobic component to the T3SS needle, presumably insuring the continuum between the hollow needle and the translocon. In the case of Enteropathogenic *Escherichia coli* (EPEC), the hydrophilic component EspA polymerizes into a filament connecting the T3SS needle to the translocon composed of the EspB and EspD hydrophobic proteins. Here, we identify EspA and EspD as targets of EspC, a serine protease autotransporter of Enterobacteriaceae (SPATE). We found that *in vitro*, EspC preferentially targets EspA associated with EspD, but was less efficient at proteolyzing EspA alone. Consistently, we found that EspC did not regulate EspA filaments at the surface of primed bacteria that was devoid of EspD, but controlled the levels of EspD and EspA secreted *in vitro* or upon cell contact. While still proficient for T3SS-mediated injection of bacterial effectors and cytoskeletal reorganization, an *espC* mutant showed increased levels of cell-associated EspA and EspD, as well as increased pore formation activity associated with cytotoxicity. EspP from enterohaemorrhagic *E*. *coli* (EHEC) also targeted translocator components and its activity was interchangeable with that of EspC, suggesting a common and important function of these SPATEs. These findings reveal a novel regulatory mechanism of T3SS-mediated pore formation and cytotoxicity control during EPEC/EHEC infection.

## Author Summary

Enteropathogenic *Escherichia coli* (EPEC) is an important diarrheal pathogen responsible for infant diarrhoea associated with significant morbidity and mortality rates in developing countries. Upon ingestion EPEC colonizes the intestinal mucosa, causing characteristic lesions on enterocytes. Using a type III secretion system (T3SS) acting as a molecular syringe, EPEC injects numerous bacterial proteins into host cells that disrupt the intestinal epithelium homeostasis. Injection of T3SS proteins requires the insertion into the host cell plasma membrane of bacterial protein complex, called the "translocon", associated with pore-forming activity. In addition to the T3SS, EPEC also secretes other bacterial toxins involved in virulence. Among these, the EspC is a protease reported to degrade various host proteins. In this paper, we have characterized an "unsuspected role" for EspC. We show that EspC degrades the T3SS translocon components following cell contact and regulates T3SS-dependent pore formation in epithelial cells. The EspC control of pore formation limits cytotoxicity and thus, is expected to limit the emission of danger signals, which would otherwise favour bacterial clearance at the onset of infection. This work describes a novel regulatory mechanism of pore formation mediated by the T3SS, that are likely to be relevant for other extracellular pathogens.

## Introduction

EPEC and EHEC are related pathogens causing severe diarrhoeal diseases. EPEC and EHEC form Attaching and Effacing (A/E) lesions on the mucosal intestinal surface, corresponding to the destruction of enterocyte microvilli and the intimate bacterial adherence to the host cell plasma membrane onto an actin-rich pedestal structure [1]. A/E pathogens carry the Locus of Enterocyte Effacement (LEE) encoding a type III secretion apparatus (T3SA) that allows the delivery of bacterial effector proteins directly from the bacterial cytoplasm into the cytoplasm of eukaryotic cells [2]. The translocator proteins EspA, B and D are required for the injection of type III effectors. Upon cell contact, EspB and EspD insert into the host cell plasma membrane and associate into a pore-forming “translocon” complex. The hydrophilic translocator protein EspA polymerizes into a hollow filamentous structure connecting the T3SA needle to the translocon [3].

SPATEs are serine protease autotransporters that are widely spread among Enterobacteriaceae. SPATEs have been reported to cleave host proteins implicated in diverse functions [4,5]. EspC has been described to cleave focal adhesion proteins, following cellular internalization, as well as other eukaryotic proteins such as haemoglobin, pepsin and human coagulation factor V [5,6,7]. Epidemiological studies indicated that EspC was predominantly found in t-EPEC strains and that EPEC strains carrying EspC and the OI-122 pathogenicity island were associated with high virulence [8,9]. These observations suggest that EspC could contribute to bacterial virulence by regulating the action of virulence factors.

Although secretion of EspC occurs through a T3SS-independent mechanism, several intriguing features link EspC and the T3SS. The expression of EspC is coupled to that of the T3SS, and secretion of EspC is also activated upon cell contact [10,11,12]. In addition, although the underlying mechanism is unclear, efficient uptake of EspC by host cells requires the T3SS [13].

These observations prompted us to investigate functional links between EspC and the T3SS. Here, we provide evidence that EspC cleaves the translocon components, EspD and EspA. EspC proteolytic activity regulates pore formation mediated by the T3SS to prevent cytotoxicity during bacterial infection.

## Results

### EspC-dependent degradation of the EspD and EspA translocators components

**Fig 1:**
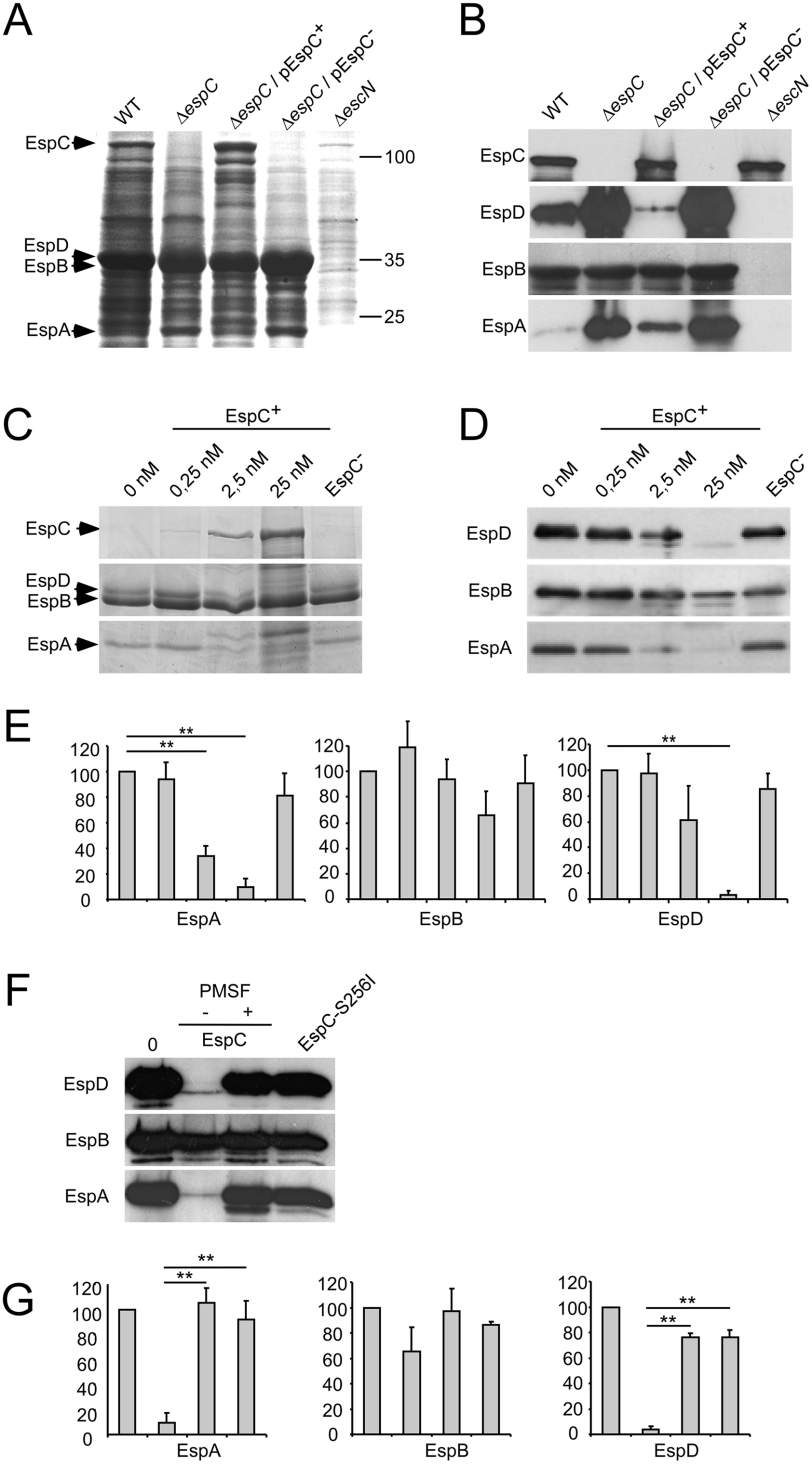
The T3SS translocator components EspA and EspD are proteolyzed by EspC. EPEC strains were grown for 16 h in DMEM to induce T3S. (A, B) Protein contents in supernatant of the indicated bacterial strain were analyzed by Coomassie blue staining (A), or Western blot using the antibodies indicated on the left (B). pEspC^+^ and pEspC^-^ indicate growth in inducing condition (arabinose) or repressing condition (glucose), for *espC* expression, respectively. (C, D, E) The EspB-D-A containing supernatant of mid-exponential DMEM-cultured-Δ*espC* strain was incubated for 16 h with recombinant EspC at the indicated concentrations and analysed by Coomassie blue staining (C), or Western blot using the antibodies indicated on the left (D and E). EspC^-^ indicates incubation with extract in the absence of induction. (F, G) Incubation was performed with 25 nM EspC in the presence or absence of PMSF, or with EspC-S256I. (E, G) Quantification of the protein band integrated density was performed in at least 3 independent experiments as shown in (D) and (F), respectively, using the image J software. Results are expressed as the average ± SEM (E, G). *: p ≤ 0.05; **: p ≤ 0,01.

To study T3SS targets of EspC, bacterial secretion profiles were first analyzed. Bacteria were grown in DMEM medium to induce secretion of T3 substrates and bacterial supernatants were analyzed by Coomassie staining (Experimental Procedures). Comparison of the profiles observed for wild-type (WT) E2348/69 EPEC and the *escN* mutant supernatants showed three major bands identified by mass spectrometry as type III secreted substrates EspB and EspD (forming a single band of 35 kDa) and EspA (22 kDa) (Fig 1A, arrows; Experimental Procedures), as well as EspC (110 kDa), an autotransported serine protease belonging to the SPATEs family. Interestingly, the EspC presence correlated with lower levels of EspB/D and EspA. Western-blot analysis showed increased levels of secreted EspA and EspD in the *espC* mutant, while the levels of EspB were similar to those observed in WT EPEC (Fig 1B). Complementation of the *espC* mutant with pEspC resulted in a reduction of EspA and EspD levels in the culture supernatant (Fig 1B). In contrast, the levels of EspB were not affected by *espC* expression. Similar results were obtained in Western blot analysis of bacterial cell fractions, showing that the levels of EspA and EspD were enhanced in the *espC* mutant and reduced in the Δ*espC* / pEspC^+^ complemented strain relative to WT (S1A Fig). In contrast to supernatants, however, the levels of EspB were also affected by EspC, consistent with association of EspB with EspA / EspD [14,15]. As expected, proteinase K treatment indicated that EspA, EspB and EspD were secreted (S1B Fig).

To test whether EspC directly regulated the levels of EspA and EspD through proteolytic degradation, supernatants from Δ*espC* mutant were incubated with recombinant EspC. Samples were analyzed by SDS-PAGE followed by Coomassie staining (Fig 1C) or by Western-blot (Fig 1D and 1E). EspA and EspD were degraded in an EspC dose-dependent manner (Fig 1C and 1D). In contrast, and consistent with results observed in the supernatant of the *espC* mutant, the EspB levels were only affected at high concentrations of EspC (Fig 1D and 1E). As shown in Fig 1F and 1G, proteolytic degradation of EspA and EspD was not observed in the presence of the serine protease inhibitor PMSF, or when EspA and EspD were incubated with EspC-S256I, a catalytic site mutant of EspC [16]. Together, these data indicate that EspC promotes the proteolysis of EspA and EspD.

### EspC preferentially targets EspA / EspD- over EspA-containing structures

**Fig 2:**
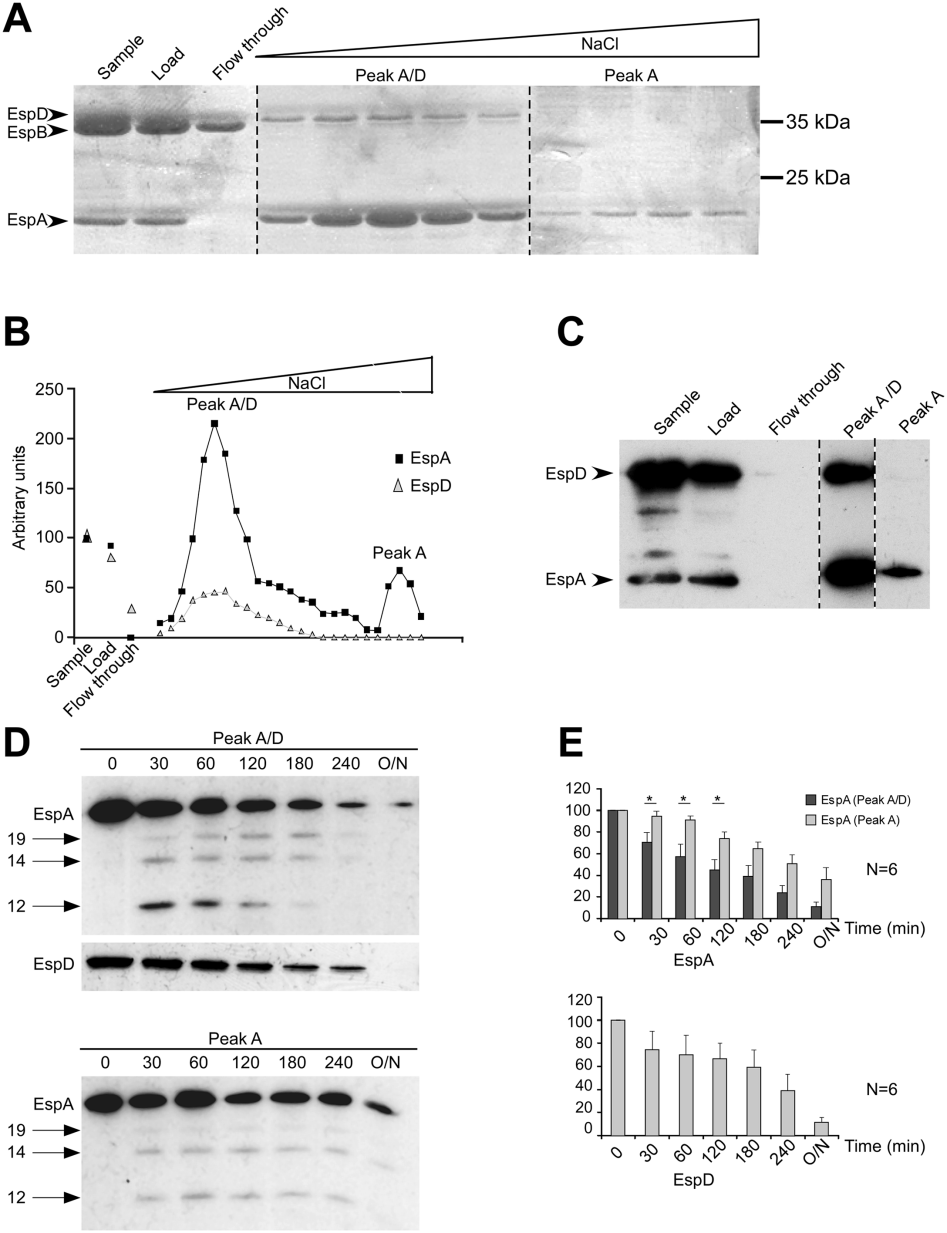
EspC preferentially targets EspA/EspD-containing structures. (A-C) Proteins from the supernatant of Δ*espC* strain were fractionated by anion exchange chromatography, using a linear NaCl gradient. (A) Fractions were analysed by Coomassie blue staining. (B) quantification of the EspA and EspD Coomassie stained bands integrated density in (A). (C) anti-EspA and anti-EspD Western blot analysis. Dashed lines indicate editing between lanes from the same gel. EspB eluted in the flow-through. The first peak containing EspA and EspD eluted at 175nM NaCl (peak A/D), the second peak containing EspA eluted at 290 mM NaCl (peak A). (D, E) Fractions corresponding to peak A or peak A/D were incubated with 40 nM of purified EspC for the indicated time. (D) Western blot analysis using the antibodies indicated on the left. Arrows indicate proteolytic degradation products observed for EspA. (E) quantification of the protein band integrated density. Results are expressed as the mean integrated density ± SEM from at least 3 independent experiments.

To characterize the activity of EspC on EspA and EspD structures, secreted proteins were fractionated by chromatography (Experimental Procedures). When subjected to anion-exchange chromatography, EspA eluted as two distinct peaks at 175 mM (peak A/D) and 290 mM NaCl (peak A) (Fig 2A and 2B). EspB did not associate with the anion exchange column under the conditions used. Interestingly, EspD co-eluted with EspA in peak A/D. Coomassie staining following SDS-PAGE indicated that EspA and EspD represented the main protein species in peak A/D, while EspA was predominant in peak A (Fig 2A and 2C).

We then tested the sensitivity of proteins contained in peaks A/D and A to EspC. To this end, EspC was purified and used at a concentration of 40 nM, which led to significant proteolysis of EspA and EspD, and which was comparable to estimated concentrations of EspC in bacterial culture supernatants (Experimental Procedures; S2A and S2B Fig). As shown in Fig 2D, EspC induced the proteolysis of EspA, with the appearance of degradation products detected by Western Blot analysis (Fig 2D arrows). While these degradation products were observed for both A/D and A fractions, proteolysis of EspA occurred steadily as a function of the incubation period with EspC in the A/D fraction (Fig 2D). In contrast, in the A fraction, EspA appeared less sensitive to EspC proteolysis during the first hour of incubation, and only showed degradation after longer incubation periods (Fig 2D). When quantified in independent experiments, EspA in the A/D fraction showed a rapid and linear decrease, while EspA decrease in the A fraction was observed at similar rate in the A fraction, but only after prolonged incubation (Fig 2E). In the A/D fraction, as for EspA, EspD was also efficiently proteolyzed by purified EspC (Fig 2D and 2E). Kinetic data analysis indicated that proteolysis of EspD in fraction A/D and EspA in fraction A occurred at a similar rate constant, but that proteolysis of EspA in fraction A/D occurred with a rate constant that was 1.8-fold higher (S3 Fig).

Taken together, these data show that EspC preferentially targets EspA in EspA / EspD-containing fraction associated with punctiform structures. In contrast, EspD in fraction A/D and EspA in the fraction containing EspA alone was less efficiently proteolyzed by EspC. These results suggest that EspC preferentially proteolyzes EspA in complex with EspD.

### EHEC EspP also cleaves EspA and EspD from both EPEC and EHEC

More than 20 different SPATEs have been described in pathogenic Enterobacteriaceae [17]. Phylogenetic analysis of the SPATEs revealed that the closest SPATEs from EspC are the uropathogenic *E*. *coli* Sat, the *Shigella flexneri* SepA and the EHEC EspP [5]. Sequence alignments between EspC and these related SPATEs indicated identity ranging from 29 to 49%, and from 44 to 64% for the protease and passenger domains, respectively (S4A Fig).

To test proteolytic specificity of SPATEs towards EPEC translocon components, EspA, B and D, recovered from the supernatant from DMEM-cultured Δ*espC* mutant were incubated with the SPATEs EspC, Sat, SepA or EspP, secreted from recombinant DH5-α (Experimental Procedures). As shown in S4B Fig, EspP, but not Sat or SepA, induced the proteolysis of EspA and EspD from EPEC. To investigate the role of EspP in the regulation of the levels of EHEC translocator components, an *espP* deletion mutant isogenic to the WT 85–170 EHEC strain was generated and secretion assays were conducted. Consistent with the results observed for EspC from EPEC, the *espP* mutant strain showed increased levels of EspA and EspD in the culture supernatant compared to WT EHEC, while EspB levels were not affected by *espP* expression (S4C Fig. Also, EHEC EspA and EspD were degraded upon incubation with either EspP or EspC secreted from recombinant DH5-α S4C Fig.

These results indicate that EspP and EspC are interchangeable and have a proteolytic activity towards T3SS translocator components. The absence of proteolytic activity of other SPATEs towards translocator components suggest that this activity of EspC / EspP is specific for EPEC / EHEC.

### EspC does not regulate EspA filaments at the surface of primed bacteria

Priming in DMEM leads to the combined induction of EPEC genes, the T3SA assembly, as well as to the activation of secretion of translocon components and type III effectors in the medium. Initial events of T3S induction are expected to involve translocator components in association with the T3SA needle at the bacterial surface, either as part of a tip complex or following induction of secretion. In other well-studied systems, such as the *Shigella* T3SS, one hydrophobic translocon component associates with the hydrophilic component to form a tip complex that negatively regulates T3S in the absence of host cells [18]. Following cell contact-dependent induction of secretion, the membrane-inserted translocon is thought to remain connected to the T3SS-needle through interaction between these hydrophobic and hydrophilic components. Although such tip complex has not been visualized for EPEC, the ortholog prediction indicates that the EPEC tip complex consist of EspA and EspD that we identified as the main EspC substrates.

**Fig 3:**
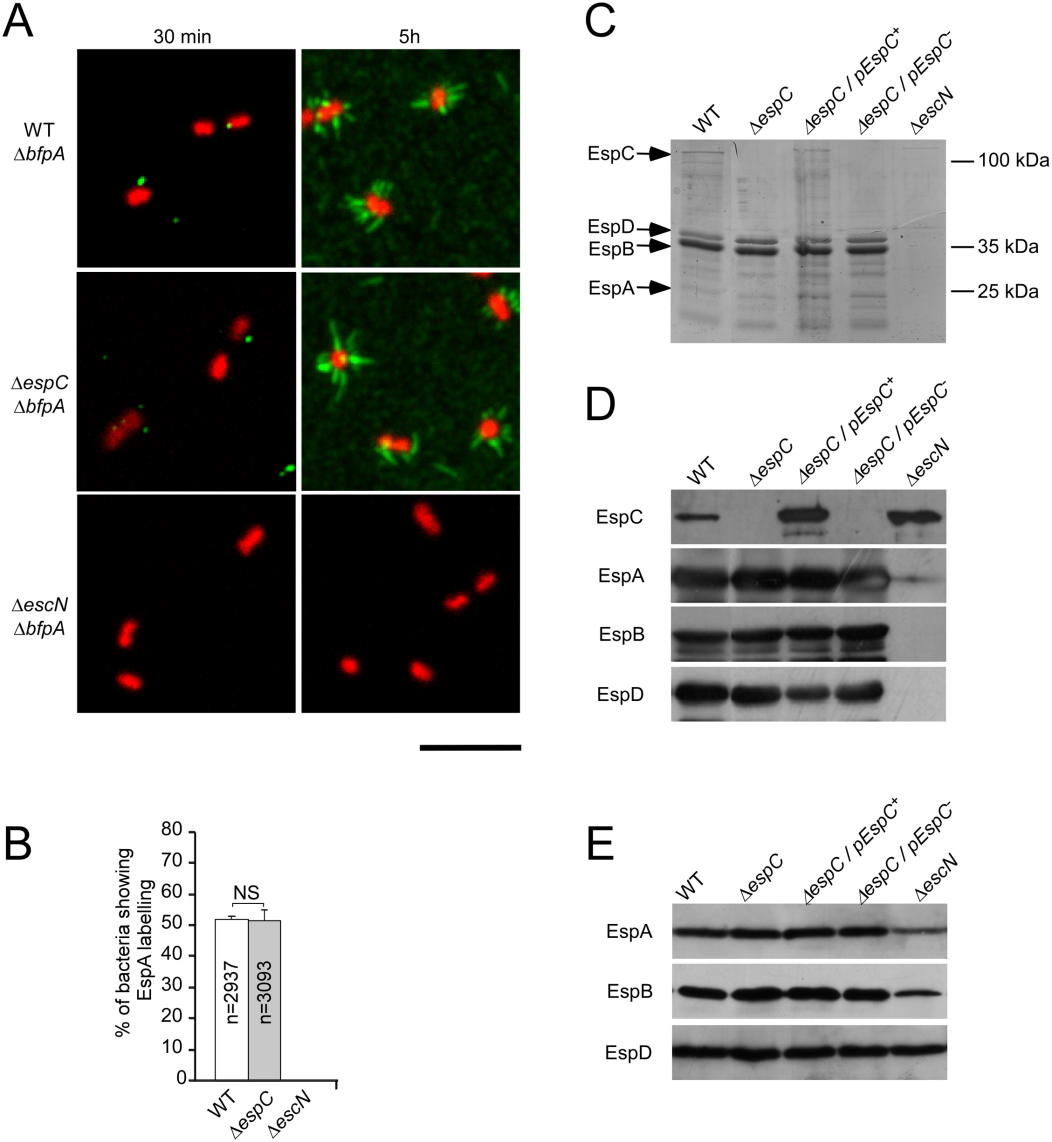
EspC does not regulate EspA filament structures at the surface of primed bacteria. EPEC strains were grown for 5 hrs in DMEM to induce T3S. (A) Epifluorescent micrographs showing EspA staining associated with bacteria primed for 30 min or 5 h in DMEM. (B) Average percentage of bacteria associated with EspA staining ± SEM, scored for at least 2900 bacteria for each sample in 3 independent experiments. The total number of analysed bacteria (n) is indicated. Scale bar = 5 μm. (C) Samples were analyzed by Coomassie blue staining. (D, E) Western blot using the antibodies indicated on the left. (C,D) bacterial supernatants; (E) bacterial pellets.

To determine if EspC regulates EspA/D in association with the T3SS, bacterial strains were primed for various time periods in cell culture medium, and protein levels were analyzed by immunofluorescent staining of bacteria immobilized onto coverslips. To unambiguously analyse structures on individual bacteria, we constructed and analyzed *bfp* mutant strains that do not form bacterial clusters (Experimental Procedures). After 30 min priming, EspA punctiform structures were occasionally detected on the bacterial surface of WT and Δ*espC* mutant strains (Fig 3A). After priming for 5 hours, EspA filaments could be readily detected at the surface of bacterial strains, suggesting that filaments evolved from punctiform structures. No difference, however was observed between WT and the Δ*espC* mutant strains, with 52 ± 1.4 and 51.5 ± 1.6% of bacteria associated with EspA filaments, respectively, with no striking difference in length and number of EspA filaments (Fig 3A and 3B). As expected, EspA labelling was not observed for an isogenic T3SS-deficient mutant Δ*escN* (Fig 3A and 3B). Western-blot analysis did not reveal EspD expression after 30 min incubation, and while EspD was detected after 5 hours incubation, no significant difference in the levels of EspA, EspB or EspD could be observed in supernatants (Fig 3C and 3D) or in bacterial pellets (Fig 3E) of WT and Δ*espC* mutant strain.

Altogether, these results indicate that EspC proteolytic activity does not regulate EspA/D structures at the bacterial surface during the 5 hours-priming in cell culture medium. Rather, EspC-mediated proteolysis appears to target EspA/D released after prolonged incubation in cell culture medium, which induces the release of translocator components normally occurring upon cell contact.

### EspC controls the amounts of EspA and EspD secreted upon cell contact

**Fig 4:**
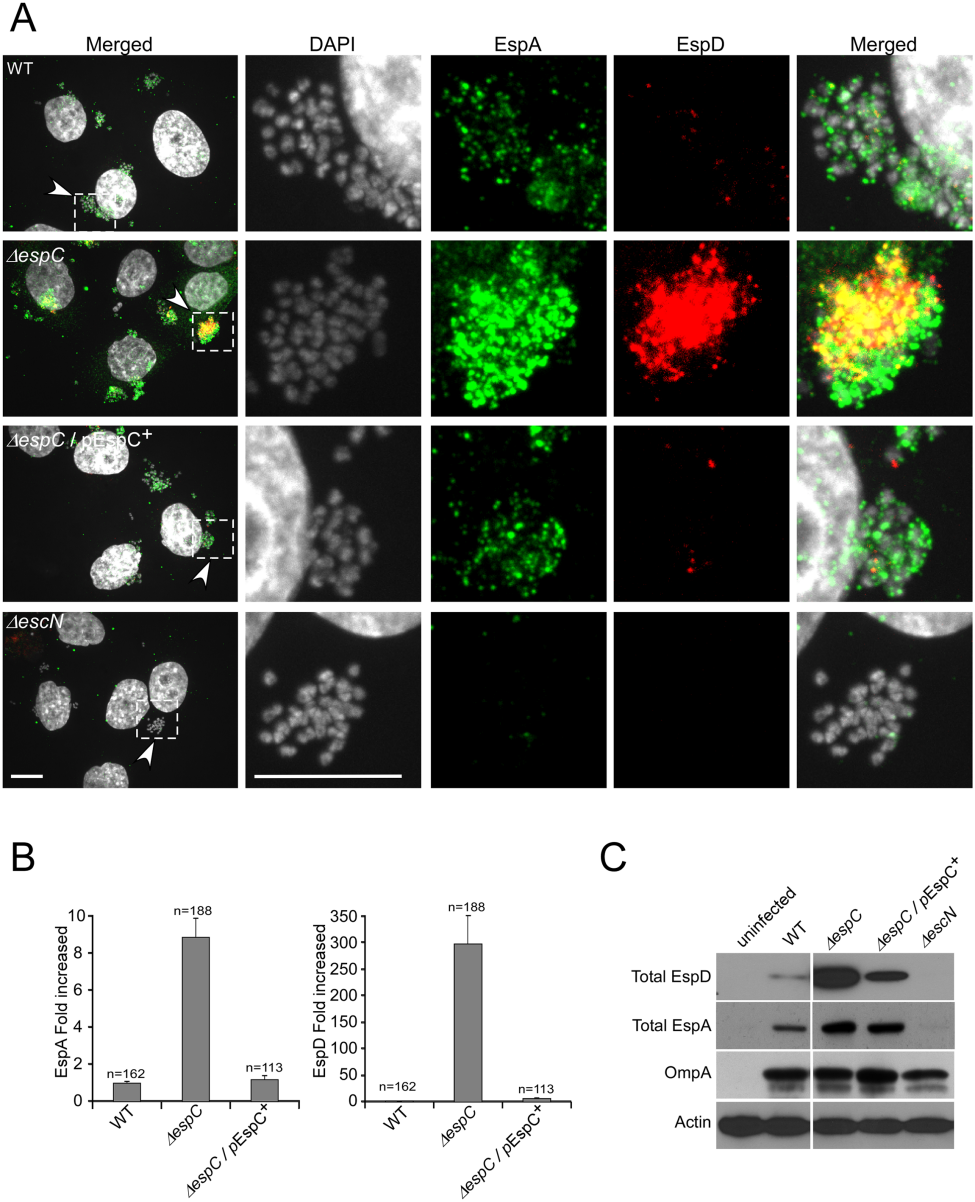
EspC negatively regulates the amounts of EspA and EspD secreted upon cell contact. (A) Confocal micrographs of cells infected for 45 min with the indicated bacteria and processed for fluorescence staining. Scale bar: 10 μm. Middle panels: magnification of insets in the corresponding left panels. Green: EspA; red: EspD staining; grey: DAPI staining. (B) Quantification of the average fluorescence intensity for EspA and EspD in microcolonies. (C) Total extracts of HeLa cells containing T3S effector proteins were subjected to anti-EspD Western Blot analysis. Anti-actin and anti-OmpA Western blotting were used as controls for cellular and bacterial loads, respectively.

While priming in culture medium triggers the assembly of the EPEC T3SA and the up-regulation of T3S substrates, it does not recapitulate the sequence of regulatory events occurring upon cell contact. For instance, cell contact has been reported to stimulate further up-regulation of EspD and EspA as well as their secretion [19,20]. To analyze the role of EspC in the regulation of EspD and EspA levels upon cell contact, immunofluorescence staining was performed on HeLa cells challenged for 45 min with primed EPEC strains (Experimental procedures). We observed EspA staining in association with all EPEC micro-colonies, bound or not to epithelial cells (Fig 4A). As described previously, EspD staining was not observed for all bacteria but whenever detected, was visualized as punctiform structures on the bacterial surface (S5B Fig; [20,21,22,23]). Interestingly, EspD staining was not observed for micro-colonies that were not associated with cells, and was observed at 45 min, but not at 15 min post-infection, consistent with cell contact triggering EspD secretion (S5A Fig). In WT EPEC, these EspD punctiform structures rarely co-localized with EspA (Fig 4A). Strikingly, the isogenic *espC* mutant showed a massive increase in EspD staining (Fig 4B), with virtually all micro-colonies showing EspD staining (Fig 4A and 4B). EspA also showed increased staining in the Δ*espC* mutant (Fig 4A and 4B), and structures showing EspD and EspA co-staining were readily observed (Fig 4A). As expected, such staining was not observed in cells infected with the T3SS-deficient Δ*escN* strain (Fig 4A).

To confirm these results, Western blot analysis was performed on lysates of HeLa cells infected with bacteria (Experimental Procedures). Consistent with EspD fluorescence staining, cells challenged with the Δ*espC* strain displayed higher amounts of secreted EspD compared to cells challenged with the WT strain (Fig 4C). As expected, EspD was not detected in lysates of cells infected with the T3SS-deficient Δ*escN* mutant strain (Fig 4C). When detergent solubilization procedures used to isolate translocon components associated with host cell membranes were used, only a minute fraction of secreted EspD was detected (S5C Fig). In these fractions, samples challenged with the Δ*espC* strain also showed higher EspD levels than those challenged with WT EPEC (S5C Fig). When cell challenge was performed with WT EPEC in presence of the serine protease inhibitor PMSF, or with the Δ*espC* mutant complemented with the protease deficient EspC-S256I, the levels of secreted EspD were comparable to those observed for the Δ*espC* mutant, consistent with the down-regulation of translocator components by EspC proteolysis (S6A and S6B Fig).

As shown in S7A and S7B Fig, when mixed infection experiments were performed using WT/GFP and Δ*espC* strains at a 1:1 ratio, heterogenous microcolonies containing variable relative amounts of these bacteria could be detected in association with cells. When quantification was performed by scoring the percentage of microcolonies associated with EspD staining, irrelevant of the EspD staining intensity, the percentage of EspD-positive microcolonies in mixed infections was similar to that observed with the *ΔespC* strain, suggesting that EspC complementation was inefficient. Consistently, immunofluorescence analysis revealed that within these mixed microcolonies, EspD staining was predominantly associated with *ΔespC* bacteria while virtually not detected with WT bacteria. These observations suggested that EspC had a more efficient cis-proteolytic activity on EspD and a poorly diffusible trans-complementation activity (S7A and S7B Fig).

These results indicate that EspC negatively regulates the levels of extracellular EspA and EspD released upon host cell contact.

### EspC controls pore formation mediated by the T3SS

We next investigated the role of EspC in T3SS-mediated cellular responses during bacterial infection. As shown in S8A Fig, the Δ*espC* mutant strain did not show detectable alteration in its ability to polymerize actin at the sites of bacterial cell contact compared to WT. Also, translocation of the Tir effector was not altered in the Δ*espC* mutant, consistent with the absence of a role for EspC in the formation of A/E lesions ([24]; S8A, S8B and S8C Fig). In addition, dispersion of micro-colonies adhering to cells was observed in the Δ*espC* mutant to a similar extent to that observed for WT bacteria (S8D Fig). These observations suggested that EspC-mediated proteolysis of translocator components did not negatively regulate type III effector translocation into host cells or EPEC-mediated actin pedestal formation.

**Fig 5:**
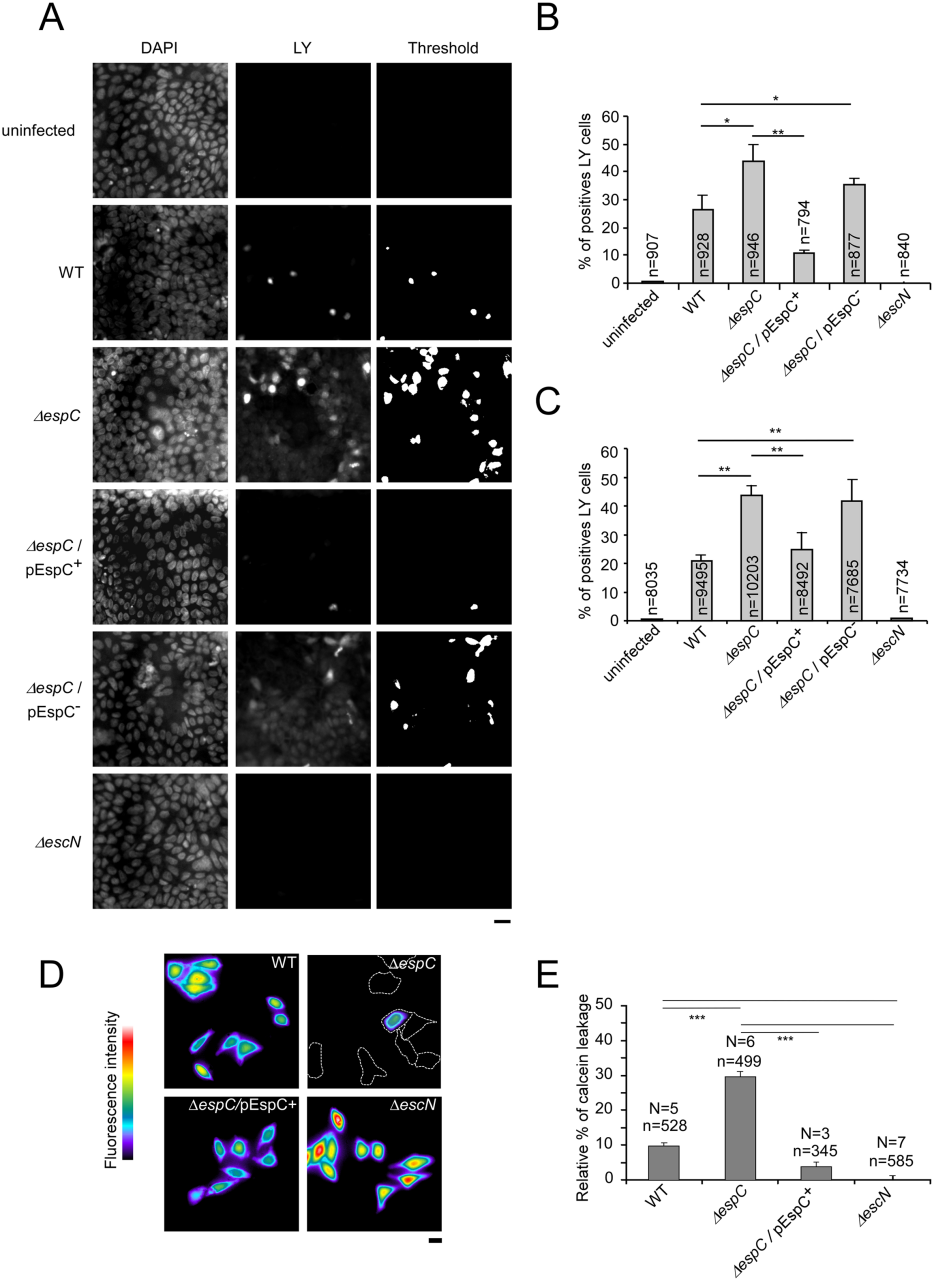
EspC controls pore formation mediated by the T3SS during cell infection. (A-C) Cells were challenged with EPEC strains for 45 min in presence of the fluorescent membrane-impermeant dye LY and analyzed by epifluorescence microscopy. (A) Representative micrographs of fixed TC7 cells showing DAPI staining (right) or LY fluorescence (middle panels). Binary images were generated by thresholding images corresponding to the LY fluorescence (right panels). LY positive cells were scored from binary images and the average percentage of LY cells / total cells ± SEM is indicated for each samples in TC7 cells (B) or HeLa cells (C). (D-E) Cells were loaded with the fluorescent dye calcein prior to bacterial challenge for 45 min. (D) Representative micrographs of pseudocolored fluorescence images of cells challenged with the indicated bacteria. Dashed lines indicate contours delineated from phase contrast images of cells with fluorescence intensity below the applied threshold. (E) The relative percentage of calcein leakage was calculated after normalization to cells challenged with the T3SS-deficient Δ*escN* strain (Experimental Procedures). The total number of analysed cells (n) and number of experiments (N) is indicated. *: p ≤ 0.05; **: p ≤ 0,01. Scale bar: 20 μm.

To analyze if EspC regulates T3SS-mediated pore formation during cell infection, we monitored the incorporation of the Lucifer Yellow (LY) fluorescent dye in cells challenged by bacteria [25,26] (Experimental Procedures, Fig 5A). As shown in Fig 5B and 5C, WT EPEC induced dye uptake in HeLa and Caco-2/TC7 cells, with 26.3 ± 5.7% and 20.9 ± 2% of cells showing LY incorporation, respectively. In contrast, no LY incorporation could be detected when cells were challenged with the T3SS-deficient mutant Δ*escN*, indicating that dye uptake was dependent on the T3SS activity. When cells were challenged with the Δ*espC* strain, however, dye uptake increased significantly, with 43.6 ± 6% and 43.5 ± 3.8% of fluorescent cells in HeLa or Caco-2 / TC7 cells, respectively. To rule out the possibility that increased uptake of LY mediated by Δ*espC* mutant strain was due to fluid-phase uptake, HeLa cells were first loaded with calcein. The decrease of calcein fluorescence mediated by pore formation in the cell plasma membrane and dye leakage was monitored following bacterial challenge for 45 min (Experimental Procedures). As shown in Fig 5D and 5E, the WT strain induced a 9.7 ± 0.9% decrease in calcein fluorescence relative to the T3SS-deficient mutant Δ*escN*. Consistent with the LY loading experiments indicative of higher pore forming activity, however, dye leakage induced by the Δ*espC* strain was significantly more pronounced relative to WT, with a 29.6 ± 1.4% decrease in calcein fluorescence (Fig 5D and 5E). The Δ*espC* / pEspC^+^ strain induced a modest 3.7 ± 1.3% decrease of dye leakage, indicative of complementation.

These results are consistent with EspC negatively regulating the formation of T3SS-dependent pores in the plasma membranes of cells during bacterial infection.

### EspC regulates cytotoxicity during bacterial infection

**Fig 6:**
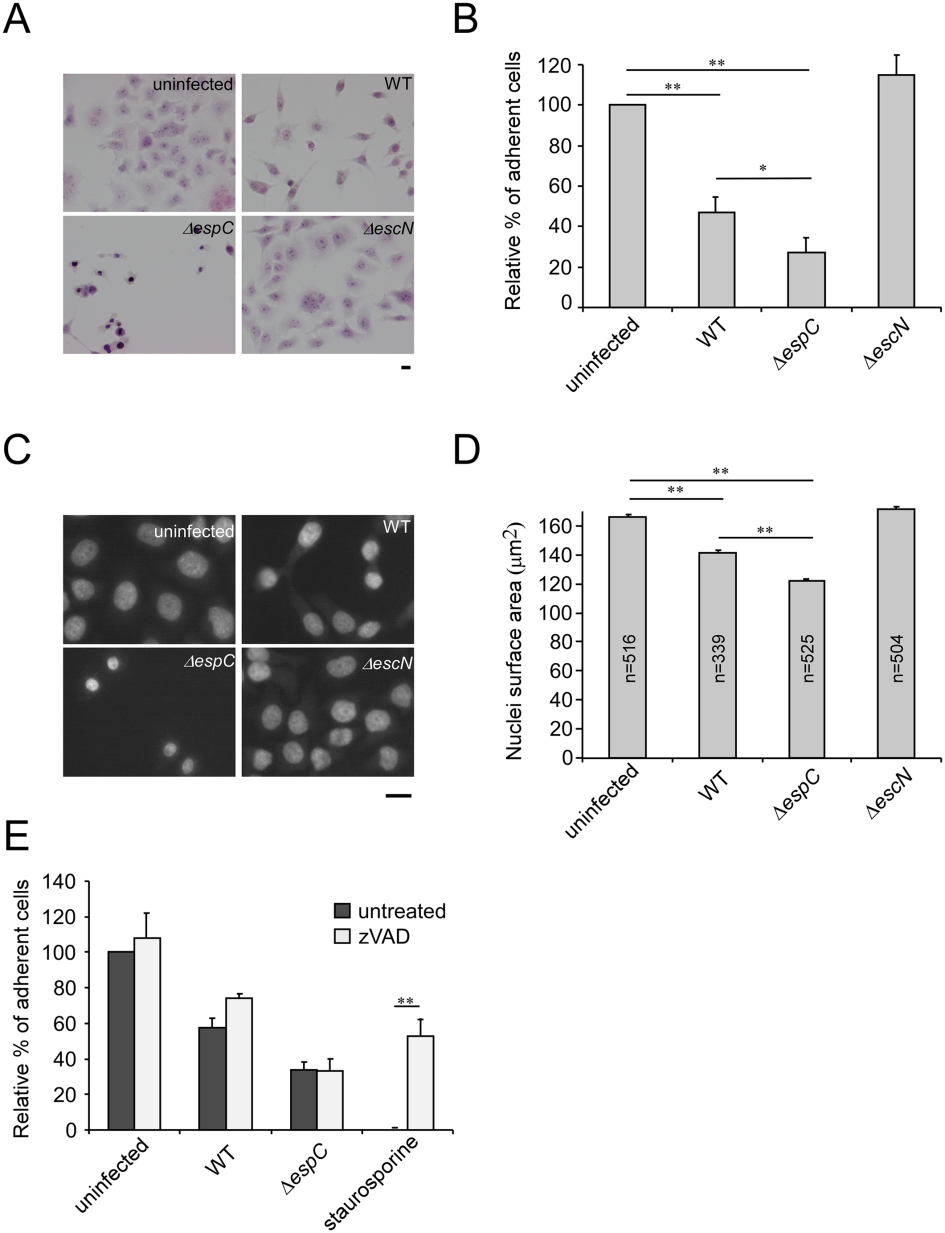
An *espC* mutant induces increased cytotoxicity. HeLa cells were challenged with EPEC strains for 45 min and infection was carried on for 18 h in presence of gentamicin (Experimental Procedures). (A) Samples were fixed and processed for hematoxylin-eosin staining. Scale bar: 15 μm. (B, E) Cells were trypsinized and scored under the microscope. The results are expressed as the average percentage ± SEM of cells relative to uninfected cells (N = 6). (C) Samples were processed for DAPI staining to visualize the cell nuclei. Scale bar: 15 μm. (D) The average surface area of nuclei ± SEM is indicated (N = 3). *: p ≤ 0.05; **: p ≤ 0,01. A T3SS-dependent cytotoxicity is observed, the *espC* mutant being significantly more cytotoxic than WT.

Host cell death can result from membrane injury induced by pore forming toxins [27,28]. Since our evidence indicated that EspC regulates the T3SS-induced pore formation in host cell membranes, we investigated whether increased pore formation associated with the *espC* mutant correlated with an increase in bacterial-induced cell death. Cells were challenged with bacteria and further incubated for 17h in the presence of gentamicin to prevent bacterial growth in the extracellular medium (Experimental Procedures). Under these conditions, bacteria-induced cytotoxicity was observed for WT EPEC, with 47.1 ± 7.3% of remaining adherent cells relative to uninfected samples (Fig 6A and 6B). Cytotoxicity was specific for the T3SS since it was not observed for the *escN* mutant (Fig 6A and 6B). Strikingly, the *espC* mutant was more cytotoxic than WT EPEC, with only 27 ± 7.7% of surviving cells recovered (Fig 6A and 6B).

Also, nuclei from *espC*-infected cells appeared more condensed than nuclei from uninfected, Δ*escN* or WT-infected cells (Fig 6A). Consistently, cells infected by the Δ*espC* mutant strain displayed an average reduction in nuclei area of 26% compared to uninfected cells (Experimental Procedures; Fig 6C and 6D). Interestingly, no DNA fragmentation was observed associated with this phenotype, suggesting that cytotoxicity was not related to caspase-dependent apoptosis. Accordingly, although bacterial infection induced detectable levels of caspase 3 cleavage that were inhibited by zVAD, this caspase inhibitor did not protect from the Δ*espC*-induced cytotoxicity (Fig 6E and S9 Fig).

## Discussion

Numerous eukaryotic targets with unrelated functions have been described for SPATEs, probably reflecting the diversity of adaptation requirements of Enterobacteriaceae to their niche [4]. Here, we describe a novel function for EspC, as a regulator of pore formation induced by the EPEC T3SS. We show that *in vitro* and during cell challenge, EspC preferentially targets EspA/EspD containing structures, and regulates the T3SS-dependent pore formation during cell challenge.

EspA was shown to interact with EspD *in vitro* [29]. By analogy with findings in other T3SS, EspA association with EspD may occur in a complex involved in host cell membrane sensing at the tip of the T3SA, which in the case of EPEC would correspond to the tip of EspA filaments. The association between EspD and EspA is also believed to establish the physical connection between the EspB/D translocon inserted into host cell membranes and the bacterial T3SA.

Our experiments did not reveal a role for EspC in the regulation of EspA/D structures at the surface of primed bacteria. Indeed, after 5-hour priming, corresponding to the priming conditions for cell infection, the Δ*espC* mutant and WT showed a similar percentage of bacteria associated with EspA filaments, with no detectable differences in EspA filament number or length. Also, similar levels of EspA and EspD were observed in the Δ*espC* mutant and WT strains. We could also detect EspA punctiform structures on the surface of bacteria primed for 30 min that are reminiscent of those observed by others [30,31]. Our time course priming studies suggest that these EspA punctiform structures were precursors of EspA filaments. In other studies, an *espD* mutant was shown to form EspA punctiform structures but failed to form EspA filaments, indicating that EspD is required for transition from EspA-containing punctiform structures to filaments [21]. The mechanism implicating EspD in this EspA-filament transition, however, remains unclear. Our preliminary results suggest that the percentage of bacteria associated with EspA punctiform structures is higher in the Δ*espC* mutant after priming for 30 min. However, it is unlikely that EspA punctiform structures are regulated through the proteolytic activity of EspC on EspA/D that we report here, since we could not detect EspD at early priming time points.

While we could not detect EspD at the bacterial surface in the absence of contact with host cells, immunostaining experiments identified EspC-sensitive EspA/D structures that were released in the supernatant of bacteria upon prolonged priming conditions. These observations suggest that if an EPEC EspA/D tip complex exists, its association with EspA filaments at the bacterial surface is too transient to allow its detection, possibly because growth in priming conditions also triggers the secretion of translocon components [32]. Alternatively, EspD may be in a configuration that is not recognized by our antibody when part of a tip complex at the bacterial surface.

Our results are consistent with EspC targeting EspA/D structures corresponding to translocator components normally released following cell contact. Mixed infection experiments indicated that within the same microcolony, EspC controls the levels of EspD associated with EspC-expressing bacteria, but not those of bacteria deficient for EspC. These results indicate that during cell infection, the action of EspC on translocator components is poorly diffusible and most efficient at the close contact of EspC-secreting bacteria. Along the same lines, the activity of EspC on EspA and EspD secreted upon cell contact appears to be more efficient than that characterized on soluble EspA and EspD released from bacteria grown *in vitro*.

Indeed, we observed a drastic increase in EspA/D levels in the Δ*espC* mutant compared to WT after 45 min following cell challenge. The T3SS-dependent pore forming activity was also increased in the Δ*espC* mutant, suggesting that EspC-mediated proteolysis of EspA/D translocator components negatively regulate the formation of translocons inserted in the host plasma membrane. Contact with host cell membranes and insertion of the T3S translocon is expected to involve a change of configuration of EspA and EspD. Following translocation, EspD is exposed on the external surface of the plasma membrane as shown by its sensitivity to degradation by proteases [20,33]. This indicates that under their membrane-inserted configuration, translocon components could also serve as targets for EspC.

The timely control of EspC-mediated degradation of translocator components suggests that EspC interacts with T3SA components prior to cell contact. Interestingly, while EspC is not a T3SS substrate, it is recovered with a high SILAC ratio in the T3SS secretome [34,35]. Furthermore, *in vitro* evidence indicates that EspC interacts with EspA [13]. Such interactions may be relevant for the control of pore formation mediated by the T3SS described in our studies. In turn, these interactions could also permit a cross-regulation of EspC activity. Indeed, EspC was reported to induce the cleavage of fodrin [7,12,36,37]. However, this intracellular fodrin cleavage activity requires the endocytosis of EspC, a process that is inefficient in the absence of a functional T3SS [37]. Once translocated, EspC may cleave fodrin, an activity linked to EPEC-induced cell detachment and cytotoxicity [7].

Promoting epithelial cell death is a dead-end for a bacterial enteropathogen, which may serves a purpose at late stages of infection and tissue colonization into a given host. The release of bacteria-containing killed cells in the intestinal lumen has been proposed to promote bacterial shedding in the environment to allow bacterial dissemination to other hosts [38]. During the initial stages of infection, along with EPEC injected type-3 effectors that down-regulate inflammation, the control of pore formation and cytotoxicity by EspC likely favours bacterial colonization of the epithelium. Consistent with a role for EspC in the negative regulation of cytotoxicity that we report here, a mutant defective for an EspC ortholog in *Citrobacter rhodentium* shows increased virulence and damaging potential observed during *in vivo* infection of mice [39].

The formation of a pore mediated by the insertion of the T3SS translocon in host cell plasma membranes is thought to be a requisite for the injection of type III effectors, and therefore probably represents a conserved feature for all T3SS. While pore-formation can be assessed in red blood cells, it is not observed in epithelial cells that are proficient for membrane-damage repair. Perhaps emphasizing the importance of their regulation, translocon-associated pores are controlled by injected type III effectors [40,41]. Such regulation may not be critical for invasive bacteria for which active translocons are expected to be removed from the plasma membrane during their inclusion in the phagocytic vacuole, but T3SS-mediated pore-formation likely represents an acute issue for extracellular pathogens such as EPEC / EHEC growing at the surface of epithelial cells. Of interest, among SPATEs expressed by T3SS-proficient enterobacteriaceae, EspP from EHEC is phylogenetically the most closely related to EspC and shares a similar protease activity towards translocon components, consistent with an adaptation of these SPATEs to extracellular growth on epithelial cells. The targeting of bacterial virulence factors by SPATEs is an emerging concept [42]. In addition to the cleavage of T3SS translocator components described in this study, EHEC EspP was reported to inactivate hemolysin (Hly) to regulate Hly-induced pore formation [43]. Obviously, a critical feature of SPATE-mediated proteolysis of pore-forming proteins is the timely control of this activity, so as not to inhibit pore formation. The study of the regulation of pore-forming activity linked to the EspA/D translocator components by EspC will likely provide insights into important steps occurring during host cell membrane's recognition by T3SSs.

## Materials and Methods

### Bacterial strains and plasmid constructs

Bacterial strains and plasmid constructs used in this study are listed in S1 Table. Bacterial culture conditions are described in S1 File.

### Cell culture

Human epithelial HeLa cells were grown at 37°C in a 5% CO_2_ incubator in RPMI (Life technology) containing Glutamax and supplemented with 10% heat-inactivated fetal calf serum (FCS) (Life Technology). TC7 cells (Caco-2/TC7) [44] were grown in a 10% CO_2_ incubator in DMEM containing 4.5 g/L glucose, supplemented with 15% heat-inactivated FCS and 1% non-essential amino acids (Life technology).

### Generation of deletion mutants

Deletions of the entire *bfpA* and *espC* genes in EPEC or *espP* gene in EHEC were created by allelic exchange using the λ Red recombinase method [45] (S1 File).

### Site directed mutagenesis

A non-proteolytic form of EspC (EspC-S256I) was generated by site directed mutagenesis on pJLM174 [35] using the Quick-change method (Stratagen, Netherlands) and the following oligonucleotides 5’- CTA CCG GTG GAG ACA TTG GTT CCG GTT TCT ATC-3’ and 5’- GAT AGA AAC CGG AAC CAA TGT CTC CAC CGG TAG-3’. The construct was verified by DNA sequencing.

### Cell infection assays

HeLa cells were plated on glass cover slips the day before the experiment at a density of 4 x 10^5^ cells for cytotoxicity assays, or 5 x 10^4^ cells for all other assays. TC7 cells were plated at a density of 7 x 10^4^ cells and allowed to polarize and differentiate for 14 days before bacterial challenge. Bacterial cultures were primed for 5 h in DMEM, centrifuged and resuspended in fresh DMEM to remove secreted proteins. Bacteria were used at a final OD_600nm_ of 0.8 for dye loading or translocation assays, or 0.05 for immunofluorescence analysis. For dye loading experiments, samples were centrifuged for 3 min at 2000 g using a plate-holder in a swinging bucket centrifuge 5810 (Eppendorf) prior to incubation at 37°C to synchronize the infection.

### Immunostaining and image analysis

Immunostaining was performed as described in the Supplemental information. Samples were mounted in Dako mounting medium (DAKO) and analysed using an Eclipse Ti microscope (Nikon) equipped with a 100 x objective, a CSU-X1 spinning disk confocal head (Yokogawa), and a Coolsnap HQ2 camera (Roper Scientific Instruments), controlled by the Metamorph 7.7 software. Analysis by epifluorescence microscopy was performed using a DMRIBe microscope (LEICA microsystems) using 380 nm, 470 nm, or 546 nm LED source excitation, equipped with a Cascade 512 camera (Roper Scientific) driven by the Metamorph 7.7 software. Images were analyzed using the Metamorph software. The levels of EspA and EspD secreted at bacterial-cell contact sites were quantified by delimiting an area corresponding to the bacterial microcolony according to DAPI staining. The integrated fluorescence intensity of relative EspA/EspD staining in the corresponding area was measured and expressed as a ratio to that obtained for DAPI staining.

### Preparation and analysis of secreted proteins

Bacteria were grown for 5h or 16 h in DMEM. The equivalent of 10 mls of bacterial culture were centrifuged at 1500 g for 15 min at 4°C. Supernatants were filtered-sterilized using a 0.22 μpore-sized filter, and proteins were subjected to precipitation using trichloroacetic acid (TCA) at a 5% final concentration and analyzed by SDS-PAGE followed by Coomassie blue staining and / or by Western Blot using the ECL-Plus detection system (Western Lighting Plus-ECL, Perkin-Elmer). Protein loading was normalized using anti-OmpA Western blotting. Quantification of the protein band integrated density was performed using the ImageJ software and expressed as the average of at least 3 independent experiments.

### Proteins purification

To purify EspC, strain DH5-α (pJLM174) was grown for 16 h in LB medium plus arabinose (0.2%, wt/vol) and ampicillin (100 μg/ml) at 37°C with shaking. Following centrifugation at 6,000 x *g* for 15 min to remove bacteria, supernatants were filtered through 0.22-μm-pore-size filters (Stericup, Millipore). Proteins from sterilized supernatant were precipitated using 40% (wt/vol) ammonium sulfate at 4°C. Pellets were resuspended in 25 mM Tris-HCl (pH 7.4), 25 mM NaCl, and 1 mM β-mercaptoethanol (βME) in a volume equal to 1/50 of the supernatant and dialyzed 3 times against 100 volumes of the same buffer. EspC was purified by FPLC using an anion exchange column (Mono Q, GE Healthcare) using a 25 mM–1000 mM NaCl 20 mls—linear gradient. Fractions were analyzed by SDS-PAGE and Coomassie staining. EspC eluted as a single peak at 265 mM NaCl. EspC-containing fraction were pooled, dialyzed against 25 mM Tris-HCl (pH 7.4), 50 mM NaCl,1 mM βME. Protein concentration was estimated using a spectrophotometer (Nanodrop, ND-1000). In typical experiments, about 3 mg of purified EspC could be obtained per millilitre of bacterial culture.

EspA and EspD were purified from the supernatant of a DMEM-cultured Δ*espC* strain, using anion exchange chromatography in a procedure similar to that of EspC, except that proteins were resuspended in 50 mM Tris-HCl (pH 7.4), 50 mM NaCl,1 mM βME in a volume equal to 1/200 of the supernatant and dialyzed against same buffer. Under these conditions, EspB did not bind to anion exchange column. EspA and EspD co-eluted at 174 mM NaCl (peak A/D), another EspA fraction free of EspD was eluted at 290 mM NaCl (peak A). Proteins were dialysed against 15 mM Tris-HCl (pH 7.4), 115 mM NaCl and 1 mM βME. The concentration of the various protein species was estimated by densitometry following SDS-PAGE and Coomassie blue staining, using purified BSA to perform standard curves.

### SPATE proteolysis assay

The EspC, EspC-S256I, Sat, SepA and EspP were expressed and recovered in the supernatant of DH5-α recombinant strains as the major protein species detected by Coomassie staining. For each SPATE, the protein concentration was adjusted to 25 nM. The SPATE-containing supernatants were incubated with the Δ*espC* strain supernatant prepared as described above, for 16 hours at 37°C. When specified, PMSF was added during the incubation procedure at a final concentration of 2 mM. Samples were TCA-precipitated and analyzed by Western Blot. For controlled proteolysis, purified EspA, EspD and EspC were used at a final concentration of 65 nM 250 nM and 40 nM, respectively, in 15 mM Tris-HCl (pH 7.4), 115 mM NaCl, 2 mM MgCl_2_ and 1 mM βME. Reactions were carried out at 37°C for different time periods (30, 60, 120, 240, 360 min or 16 hours), and stopped by the addition of Laemmli sample loading buffer and boiling for 10 min. Samples were analysed by Western Blot.

### Lucifer yellow incorporation

Cells grown onto glass coverslips were challenged with primed EPEC strains. Samples were incubated in DMEM containing 1 mM of Lucifer yellow in a wet chamber at 37°C for 15 or 45 min. In control experiments, samples were incubated with 1 mM 70 kDa dextran coupled to rhodamine. Samples were washed with PBS and fixed in 3.7% PFA. Samples were labelled with DAPI and analyzed using an inverted epifluorescence microscope. Images corresponding to LY fluorescence were thresholded to generate binary images. LY positive cells were scored from binary images and results were expressed as the average percentage ± SEM of LY positives cells relative to total cells. Different samples within the same experiment were treated in the same conditions during image acquisition and analysis.

### Calcein leakage assay

Cells grown on coverslips were loaded with 3 μM calcein-AM (Life Technology) for 30 min in EM buffer (HEPES 25 mM pH 7,3; NaCl 120 mM; CaCl_2_ 1.8 mM; MgCl_2_ 0.8 mM; KCl 7 mM; glucose 5 mM), at 21°C. After three washes in EM buffer, cells were incubated for another 30 min at 21°C. Cells were challenged with primed EPEC strains for 45 min. Samples were mounted in an observation chamber on a plate heated at 37 °C on an inverted epifluorescence microscope. For each experiment, image acquisition was performed using identical conditions for each sample. The percentage of calcein leakage was calculated as 100 x (1 - I / I_*ΔescN*_), with I corresponding to the averaged integrated fluorescence intensities of individual cells for the sample, and I_*ΔescN*_ corresponding to that of cells infected with the T3SS-deficient Δ*escN* mutant strain.

### Cytotoxicity assay and nuclear shrinkage

Bacterial strains were primed in DMEM for 5 h, and HeLa cells were infected with primed bacteria for 45 min at 37°C. The medium was removed and samples were further incubated for one hour in fresh medium. Gentamicin was added to kill bacteria and samples were incubated for 17h. Samples were fixed with 3.7% PFA and analyzed by transmission light microscopy following hematoxylin / eosin staining, or epifluorescence microscopy for DAPI staining. Alternatively, samples were washed 3 times with PBS, trypsinized and resuspended cells were scored using a Malassez cell counting chamber.

To analyze nuclear shrinkage, the nuclei area was delimited by thresholding images acquired from DAPI stained samples to generate binary images. The nuclei size was analyzed using the Metamorph software. Samples within the same experiment were treated under the same conditions during image acquisition and analysis.

When specified, the caspase inhibitor zVAD was added at a 50 μM final concentration for 30 minutes prior to bacterial challenge and maintained throughout the experiment. Staurosporine was used at 1 μM as a positive control for caspase-activation.

### Statistical analysis

Statistical analysis was performed using an unpaired Student’s t-test with unequal variance.

## Supporting Information

S1 Fig EspC controls the levels of EspA, EspB and EspD associated with the bacterial pellets. EPEC strains were grown overnight in DMEM to induce T3S. Bacterial pellets were analyzed by Western blot using the antibodies indicated on the left (A and B). **(A)** OmpA was used as a control for bacterial load. **(B)** "+" and "-" indicate Proteinase K (PK) treatment prior to Western-blot analysis. The sensitivity to PK indicated that the majority of EspA, EspB and EspD insoluble pools were secreted. Note that as opposed to secreted soluble EspB, the levels of bacteria-associated EspB depended on EspC, consistent with EspB association with the T3SS through interaction with EspD.
(TIF)

S2 Fig EspC-mediated proteolysis of EspA and EspD at steady state conditions. **(A, B)** Samples from peaks A and A/D were incubated for 16 hours at 37°C with the concentration of EspC indicated in nM, and analysed by Western blotting. **(A)** Western-blotting analysis of a representative experiment, using antibody directed against the protein indicated on the left. **(B)** Integrated density of bands detected in (A), expressed as a percentage of the indicated protein species in samples treated with buffer alone. Values are expressed as the average ± SEM of 6 independent experiments. Bars: EspA in peak A (solid), EspA in peak A/D (empty), EspD (grey). EspA as well as EspD show a dose—dependent proteolysis by EspC.
(TIF)

S3 Fig Kinetic data of EspA and EspD proteolysis by EspC. EspA (65 nM) from the A (**A**, empty circles) or A/D fractions (**A**, solid circles), or EspD (250 nM) (**B**, solid squares) were incubated with EspC (40 nM) at 37°C for the indicated time points. The relative concentration of substrate ([S] / [S]_]0_) was calculated from the density of the electrophoretic bands corresponding to EspA or EspD in Western-blot analysis, as shown in Fig 2. The ln [S] / [S]_]0_ was plotted as a function of time. The rate constants inferred from the straight lines corresponded to k_0_ = 4.7 x 10^-5^ s^-1^, 8.7 x 10^-5^ s^-1^, and 4.7 x 10^-5^ s^-1^, for EspC-mediated proteolysis of EspA in fraction A, EspA in fraction A/D, and EspD, respectively. When analyzed in terms of Michaelis-Menten kinetics and assuming K_m_ >> S_0_, k_0_ is proportional to the specificity constant k_cat_/ K_m_, with k_0_ = C_0_ x k_cat_ / K_m_, C_0_ being the initial enzyme concentration. The deduced specificity constants k_cat_ / K_m_ for EspC-mediated proteolysis of EspA in fraction A, EspA in fraction A/D, and EspD, were 1.2 x 10^3^ M^-1^. s^-1^, 2.2 x 10^3^ M^-1^. s^-1^, and 1.2 x 10^3^ M^-1^. s^-1^, respectively.
(TIF)

S4 Fig EHEC EspP also targets EspA and EspD. **(A)** Schematic representation of EspC domains. The percentages of amino acid identity or similarity (numbers between brackets) are indicated for the whole passenger domain or the protease domain of the related SPATES Sat, SepA and EspP. **(B)** EspA, EspB and EspD secreted from Δ*espC* were assayed for *in vitro* degradation by EspC, Sat, SepA or EspP (Supplementary Procedures). EspC and EspP, but not Sat or SepA, have a proteolytic activity towards EspA and EspD. **(C)** Bacterial supernatants of EHEC strains primed in DMEM were analyzed by Western blotting using the indicated antibodies. Supernatant of: wild-type EHEC (WT); *espP* mutant (Δ*espP*); Δ*espP* supernatant was incubated with 25 nM of recombinant EspP (Δ*espP* + EspP) or EspC (Δ*espP* +EspC). EspA and EspD from EHEC are sensitive to EspP as well as EspC.
(TIF)

S5 Fig The Δ*espC* mutant shows increased EspD staining during cell challenge. HeLa cells were challenged for 45 min with bacterial strains previously primed for 5 hours in DMEM. **(A, B)** Samples were fixed and processed for immunofluorescent staining of EspD (red) and bacteria (blue). Representative micrographs of cells infected with the bacterial strain indicated on the left. **(A)** Cell infected by the Δ*espC* strain. Green: F-actin. Note that EspD staining was detected for cell-associated bacteria (arrowhead) but not for bacteria not associated to cells (arrow). Scale bar: 5 μm. **(B)** Higher amounts of EspD was observed for the Δ*espC* and the MAS111 strains deficient for EspC in comparison to WT. Scale bar: 10 μm. **(C)** Cell lysates were treated with Triton X-100 to extract membrane proteins and subjected to Western Blot analysis using anti-EspD or anti-actin antibodies. Total: total lysates; Tx: Triton-X100 soluble fractions. Higher amounts of EspD were observed for the Δ*espC* compared to WT (Experimental Procedures).
(TIF)

S6 Fig EspC proteolytic activity controls the levels of EspD upon cell challenge. HeLa cells were challenged for 45 min with bacterial strains previously primed for 5 hours in DMEM. **(A)** Total cell lysates were subjected to anti-EspD Western Blot analysis. **(B)** Samples were fixed and processed for immunofluorescent staining of EspD (red) and bacteria (blue). Higher amounts of EspD were observed for the *ΔespC*, *ΔespC* complemented with a mutated form of EspC (*ΔespC* / pEspC-S256I), or WT strain in presence of 1mM of the serine protease inhibitor (PMSF) in comparison to WT.
(TIF)

S7 Fig EspD levels are regulated at the vicinity of EspC expressing bacteria. HeLa cells were infected with WT-GFP and the Δ*espC* mutant at a 1: 1 ratio. **(A)** Fluorescence micrographs corresponding to confocal planes of various fields showing EspD staining (red), GFP-WT (green) and DAPI staining (blue) (1–4). Mixed microcolonies of WT-GFP and Δ*espC* strains showed EspD staining at the vicinity of Δ*espC* but not WT bacteria, indicative of poor *EspC* complementation at a distance from EspC-secreting bacteria. White asterisks indicate GFP-WT bacteria. Scale bar: 5 μm. **(B)** The average percentage of microcolonies showing EspD staining was scored.
(TIF)

S8 Fig The Δ*espC* mutant forms actin pedestals and is proficient for Tir translocation. **(A)** HeLa cells were infected with primed EPEC strains for 45 min, fixed and processed for fluorescence staining of F-actin (green), Tir (red) and DNA (blue). Representative confocal micrographs of samples challenged with the strains indicated on the left. Scale bar: 5 μm. **(B)** The average percentage ± SEM of adherent bacteria showing Tir staining association. The values are representative of at least 500 adherent bacteria scored in 5 independent experiments. **(C)** HeLa cells were challenged with the bacterial strains, and whole cells lysates were subjected to Western Blot analysis using the indicated antibodies. Arrowhead: translocated Tir visualized as a larger migrating species [1]. No difference in Tir translocation could be detected between WT and Δ*espC* strains. These results indicate that EspC is dispensable for EPEC-induced actin reorganization and are consistent with previous observations that EspC does not affect signal transduction leading to A/E lesions [2]. **(D)** HeLa cells were infected with primed EPEC strains for 45 min or 5 hrs. Samples were fixed and processed for fluorescence staining of F-actin (red) and GFP-expressing-bacteria (green). Micrographs representative of at least 3 independent experiments are shown. Arrowheads indicate clusters of aggregated bacteria. Arrows show dispersed bacteria associated with actin pedestals. All strains formed tight bacterial clusters at 45 min. Bacterial dispersion was observed at 5 hrs p.i. for WT and *espC* mutant strain but not for the T3SS-deficient strain Δ*escN*. Bacterial dispersion was not observed when infection was carried out in the presence of cytochalasin D (cytoD).
(TIF)

S9 Fig The caspase-inhibitor zVAD prevents bacterial-induced cleavage of caspase-3. HeLa cells were treated with zVAD and challenged with primed EPEC strains for 45 min. Infection was carried on for 17 h in presence of gentamicin and zVAD. Samples were scraped in Laemmli sample buffer and subjected to Western Blot analysis using antibodies directed against full-length or cleaved caspase-3. Anti-actin Western-blot analysis is shown in the bottom panel as a loading control. Staurosporine was used as a positive control. Both WT and *espC* mutant strains induced caspase-3 cleavage, that was inhibited by zVAD.
(TIF)

S1 Table Bacterial strains and plasmids used in this study. (PDF)

S1 File Supporting information. (PDF)
