# Supplementary figures and images for: The Serine Protease EspC from Enteropathogenic Escherichia coli Regulates Pore Formation and Cytotoxicity Mediated by the Type III Secretion System
Source: PLoS Pathog. 2015 Jul 1;11(7):e1005013. doi: 10.1371/journal.ppat.1005013 (PMC4488501; doi:10.1371/journal.ppat.1005013)

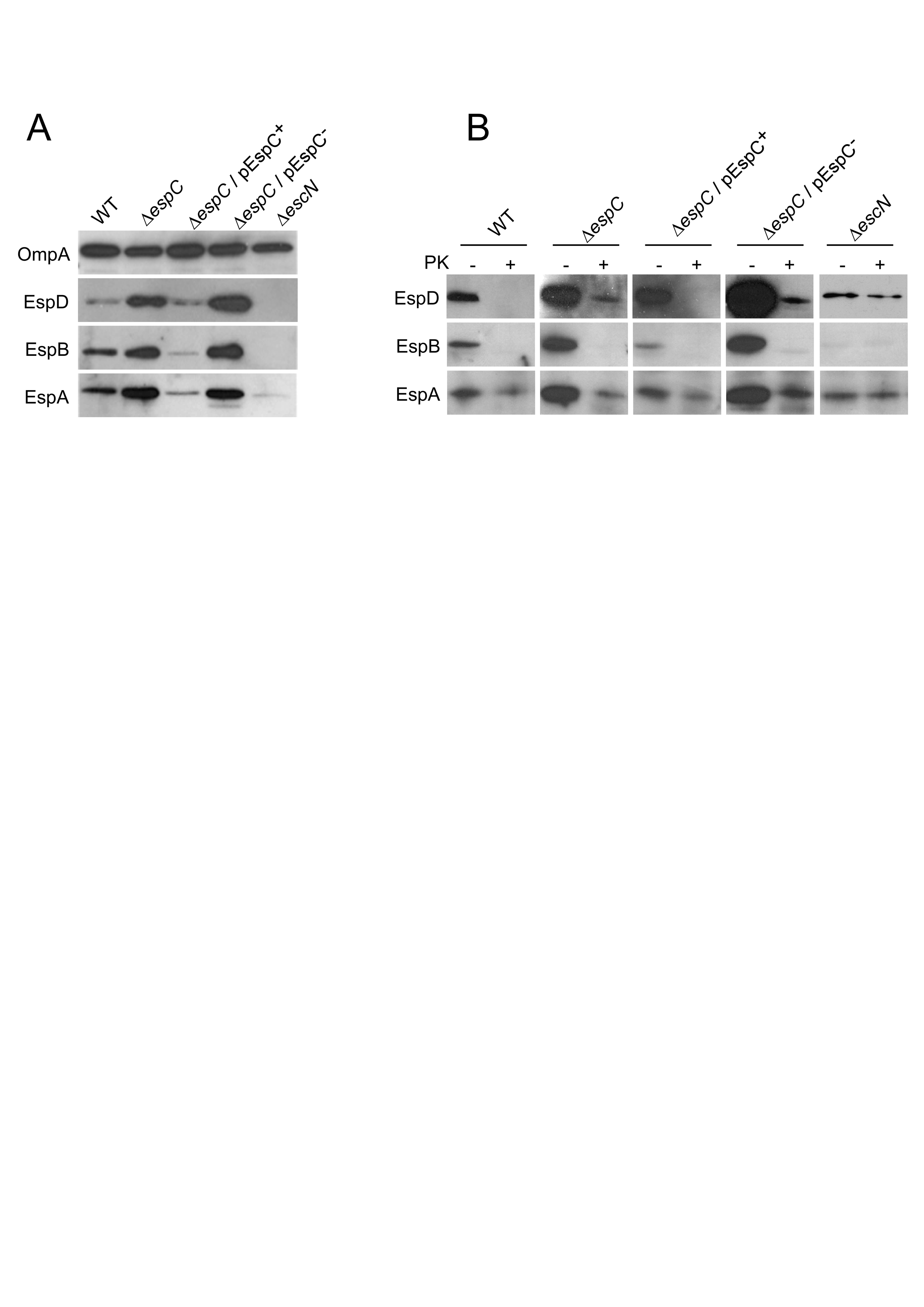

Supplement: S1 Fig — EPEC strains were grown overnight in DMEM to induce T3S. Bacterial pellets were analyzed by Western blot using the antibodies indicated on the left (A and B). (A) OmpA was used as a control for bacterial load. (B) "+" and "-" indicate Proteinase K (PK) treatment prior to Western-blot analysis. The sensitivity to PK indicated that the majority of EspA, EspB and EspD insoluble pools were secreted. Note that as opposed to secreted soluble EspB, the levels of bacteria-associated EspB depended on EspC, consistent with EspB association with the T3SS through interaction with EspD. (TIF) [file ppat.1005013.s001.tif]

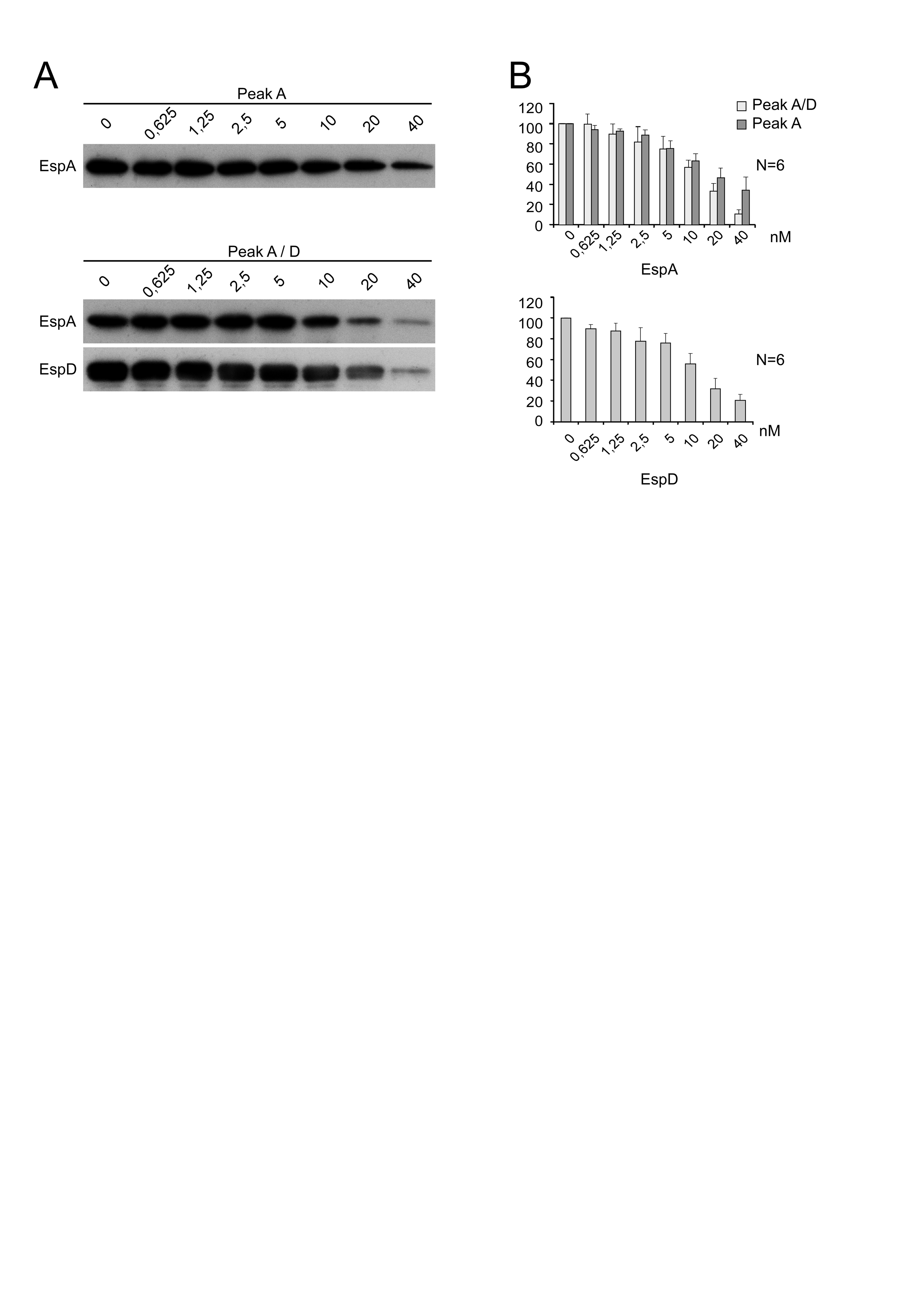

Supplement: S2 Fig — (A, B) Samples from peaks A and A/D were incubated for 16 hours at 37°C with the concentration of EspC indicated in nM, and analysed by Western blotting. (A) Western-blotting analysis of a representative experiment, using antibody directed against the protein indicated on the left. (B) Integrated density of bands detected in (A), expressed as a percentage of the indicated protein species in samples treated with buffer alone. Values are expressed as the average ± SEM of 6 independent experiments. Bars: EspA in peak A (solid), EspA in peak A/D (empty), EspD (grey). EspA as well as EspD show a dose—dependent proteolysis by EspC. (TIF) [file ppat.1005013.s002.tif]

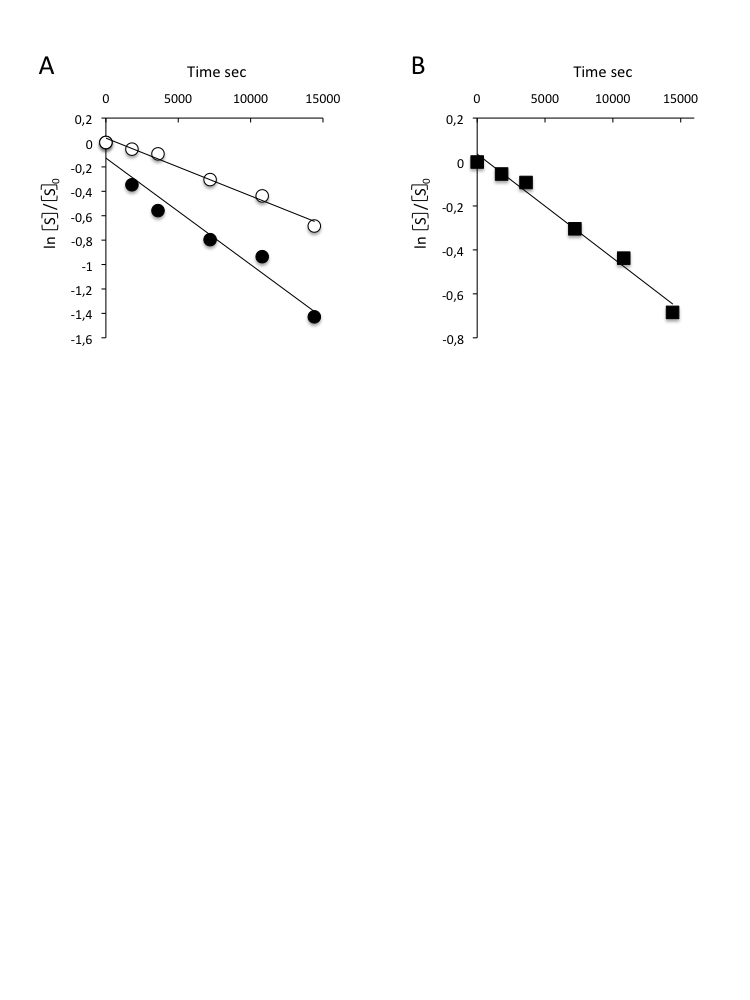

Supplement: S3 Fig — EspA (65 nM) from the A (A, empty circles) or A/D fractions (A, solid circles), or EspD (250 nM) (B, solid squares) were incubated with EspC (40 nM) at 37°C for the indicated time points. The relative concentration of substrate ([S] / [S]]0) was calculated from the density of the electrophoretic bands corresponding to EspA or EspD in Western-blot analysis, as shown in Fig 2. The ln [S] / [S]]0 was plotted as a function of time. The rate constants inferred from the straight lines corresponded to k0 = 4.7 x 10-5 s-1, 8.7 x 10-5 s-1, and 4.7 x 10-5 s-1, for EspC-mediated proteolysis of EspA in fraction A, EspA in fraction A/D, and EspD, respectively. When analyzed in terms of Michaelis-Menten kinetics and assuming Km >> S0, k0 is proportional to the specificity constant kcat/ Km, with k0 = C0 x kcat / Km, C0 being the initial enzyme concentration. The deduced specificity constants kcat / Km for EspC-mediated proteolysis of EspA in fraction A, EspA in fraction A/D, and EspD, were 1.2 x 10^3^ M-1. s-1, 2.2 x 10^3^ M-1. s-1, and 1.2 x 10^3^ M-1. s-1, respectively. (TIF) [file ppat.1005013.s003.tif]

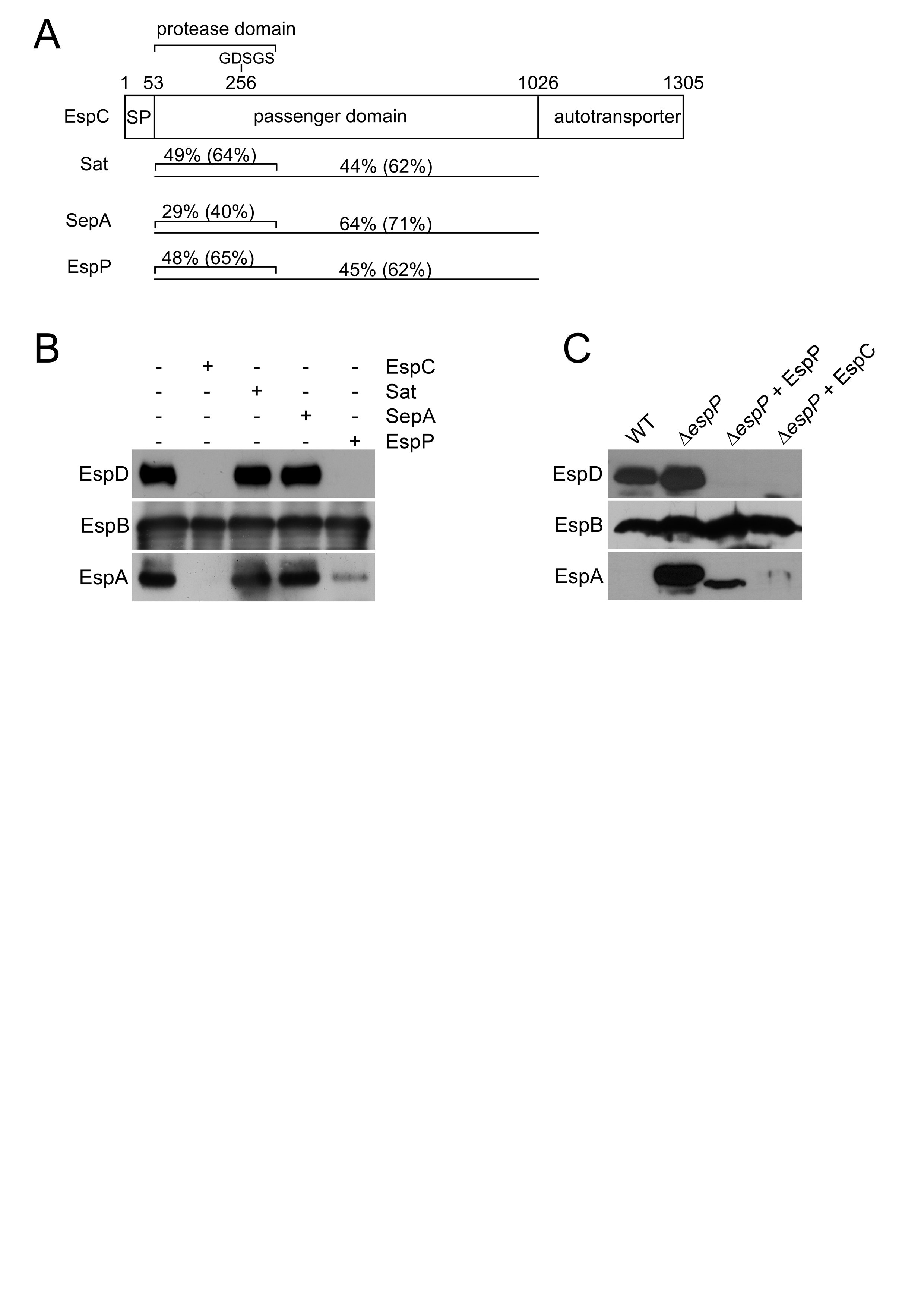

Supplement: S4 Fig — (A) Schematic representation of EspC domains. The percentages of amino acid identity or similarity (numbers between brackets) are indicated for the whole passenger domain or the protease domain of the related SPATES Sat, SepA and EspP. (B) EspA, EspB and EspD secreted from ΔespC were assayed for in vitro degradation by EspC, Sat, SepA or EspP (Supplementary Procedures). EspC and EspP, but not Sat or SepA, have a proteolytic activity towards EspA and EspD. (C) Bacterial supernatants of EHEC strains primed in DMEM were analyzed by Western blotting using the indicated antibodies. Supernatant of: wild-type EHEC (WT); espP mutant (ΔespP); ΔespP supernatant was incubated with 25 nM of recombinant EspP (ΔespP + EspP) or EspC (ΔespP +EspC). EspA and EspD from EHEC are sensitive to EspP as well as EspC. (TIF) [file ppat.1005013.s004.tif]

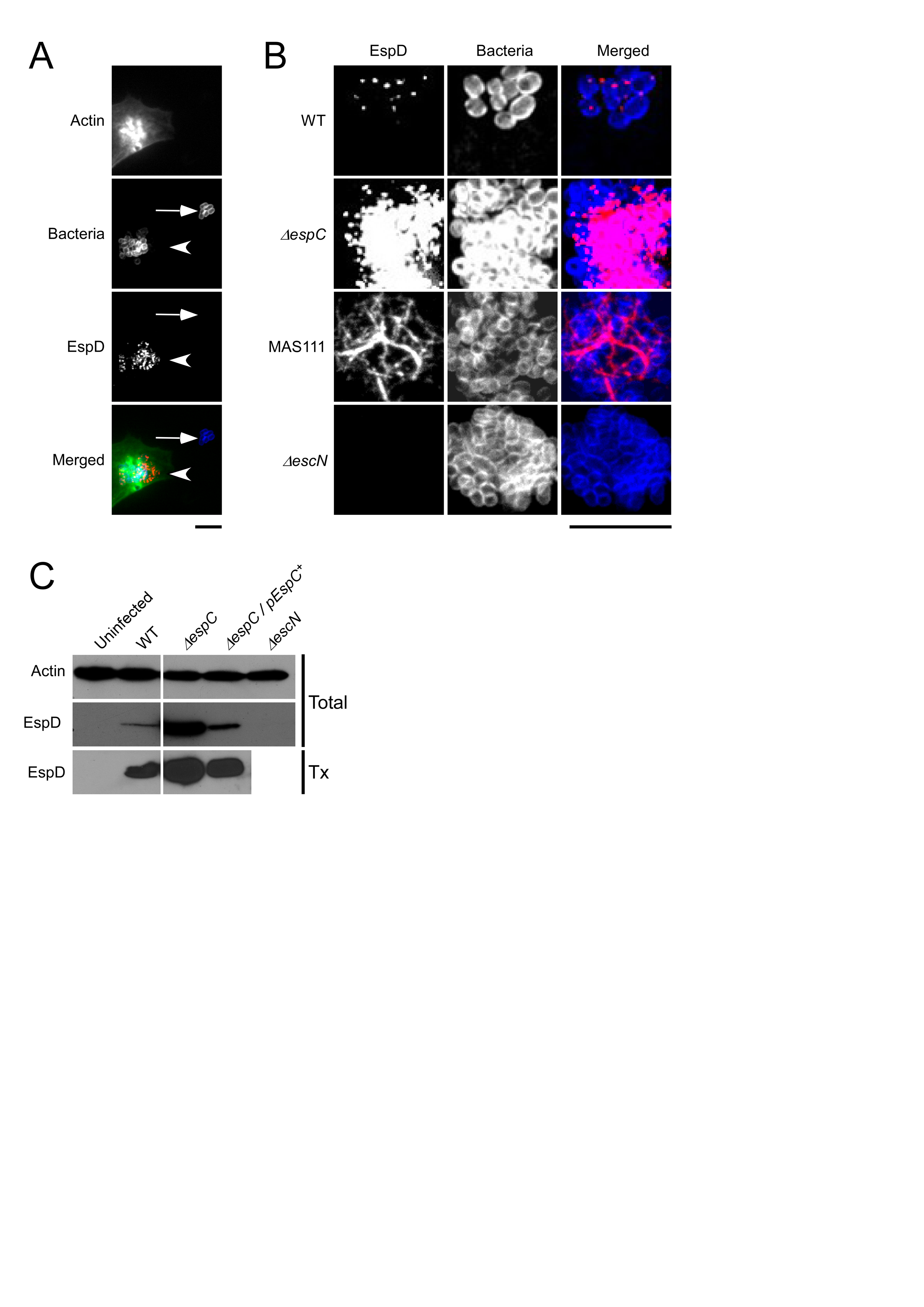

Supplement: S5 Fig — HeLa cells were challenged for 45 min with bacterial strains previously primed for 5 hours in DMEM. (A, B) Samples were fixed and processed for immunofluorescent staining of EspD (red) and bacteria (blue). Representative micrographs of cells infected with the bacterial strain indicated on the left. (A) Cell infected by the ΔespC strain. Green: F-actin. Note that EspD staining was detected for cell-associated bacteria (arrowhead) but not for bacteria not associated to cells (arrow). Scale bar: 5 μm. (B) Higher amounts of EspD was observed for the ΔespC and the MAS111 strains deficient for EspC in comparison to WT. Scale bar: 10 μm. (C) Cell lysates were treated with Triton X-100 to extract membrane proteins and subjected to Western Blot analysis using anti-EspD or anti-actin antibodies. Total: total lysates; Tx: Triton-X100 soluble fractions. Higher amounts of EspD were observed for the ΔespC compared to WT (Experimental Procedures). (TIF) [file ppat.1005013.s005.tif]

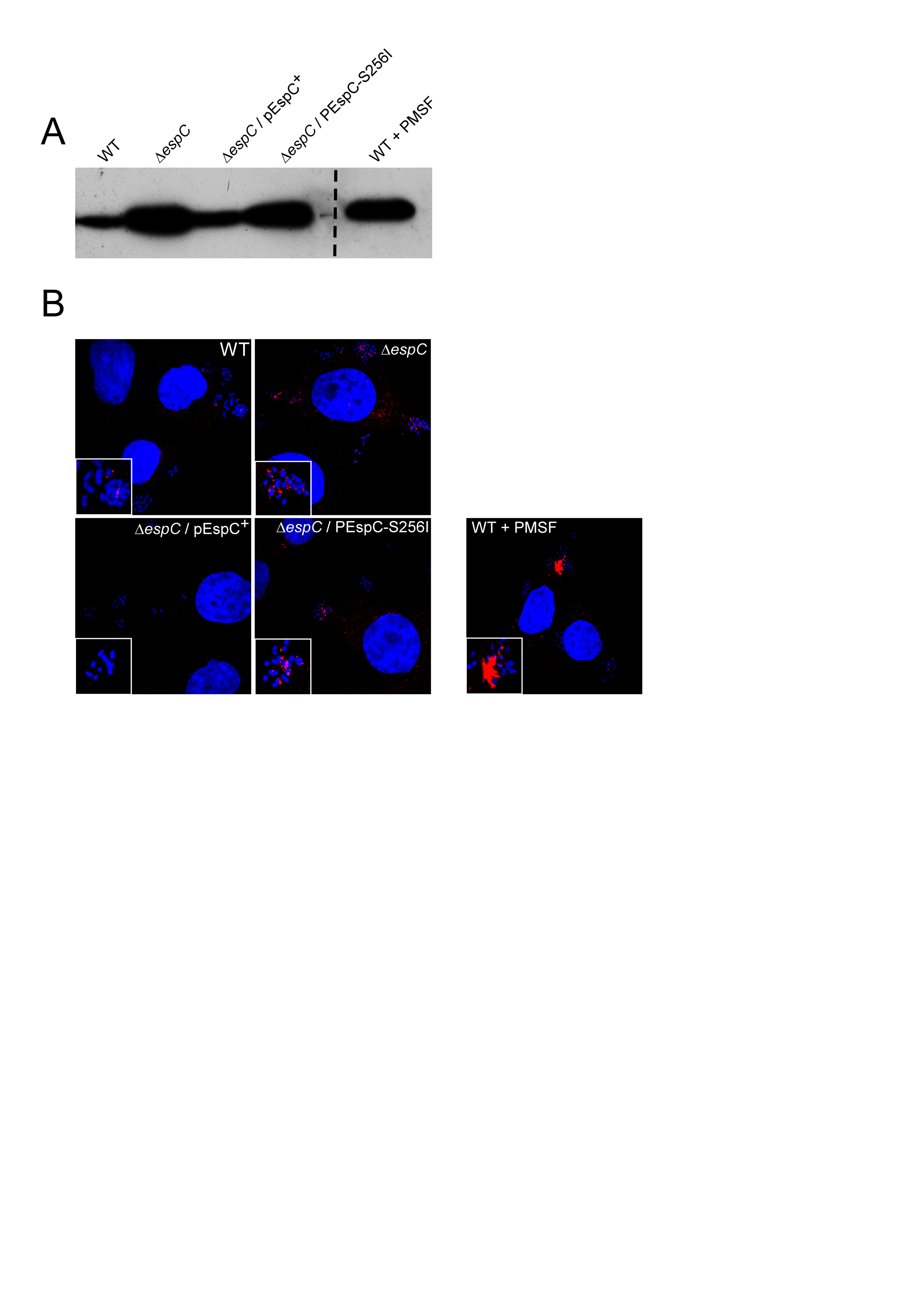

Supplement: S6 Fig — HeLa cells were challenged for 45 min with bacterial strains previously primed for 5 hours in DMEM. (A) Total cell lysates were subjected to anti-EspD Western Blot analysis. (B) Samples were fixed and processed for immunofluorescent staining of EspD (red) and bacteria (blue). Higher amounts of EspD were observed for the ΔespC, ΔespC complemented with a mutated form of EspC (ΔespC / pEspC-S256I), or WT strain in presence of 1mM of the serine protease inhibitor (PMSF) in comparison to WT. (TIF) [file ppat.1005013.s006.tif]

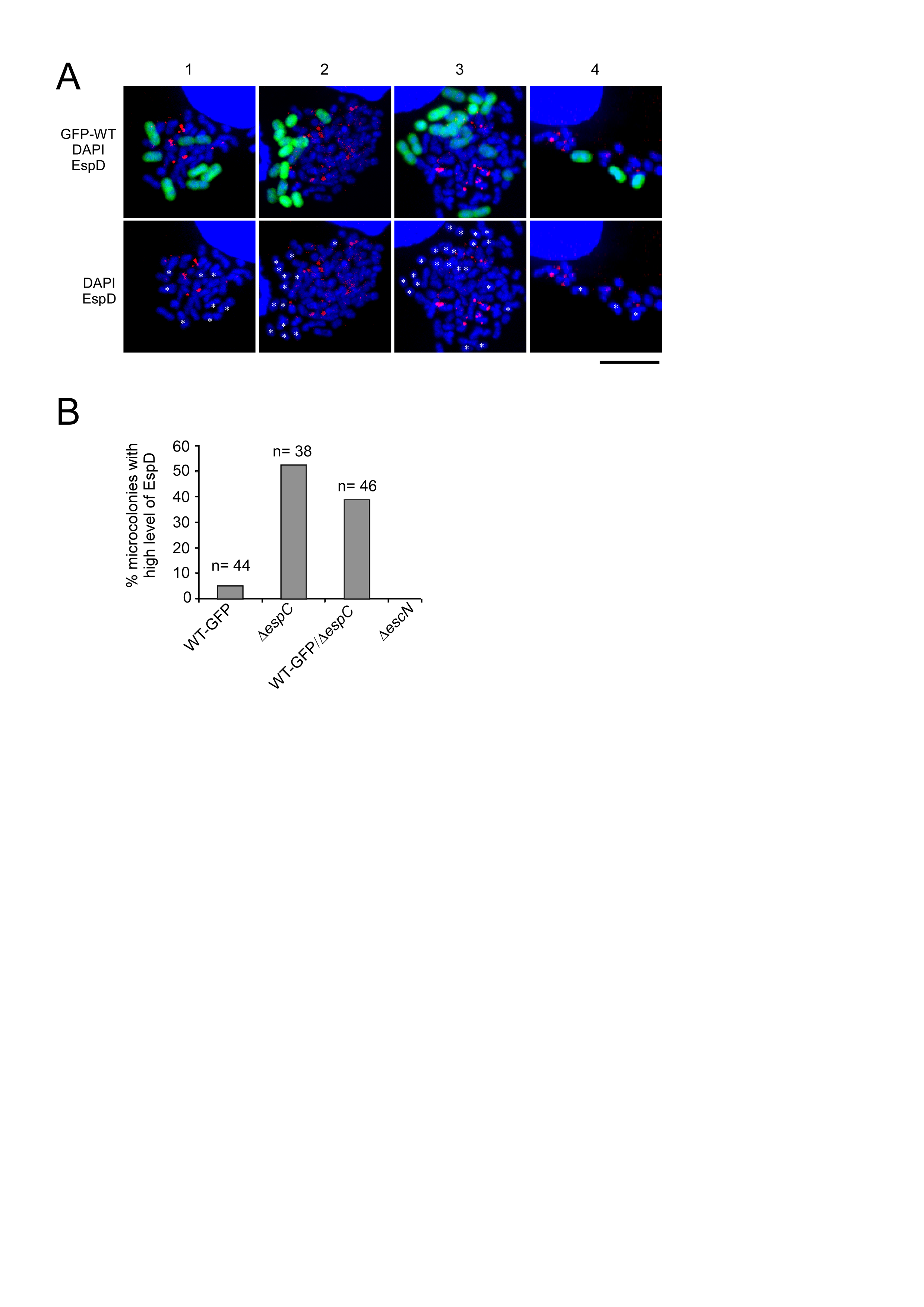

Supplement: S7 Fig — HeLa cells were infected with WT-GFP and the ΔespC mutant at a 1: 1 ratio. (A) Fluorescence micrographs corresponding to confocal planes of various fields showing EspD staining (red), GFP-WT (green) and DAPI staining (blue) (1–4). Mixed microcolonies of WT-GFP and ΔespC strains showed EspD staining at the vicinity of ΔespC but not WT bacteria, indicative of poor EspC complementation at a distance from EspC-secreting bacteria. White asterisks indicate GFP-WT bacteria. Scale bar: 5 μm. (B) The average percentage of microcolonies showing EspD staining was scored. (TIF) [file ppat.1005013.s007.tif]

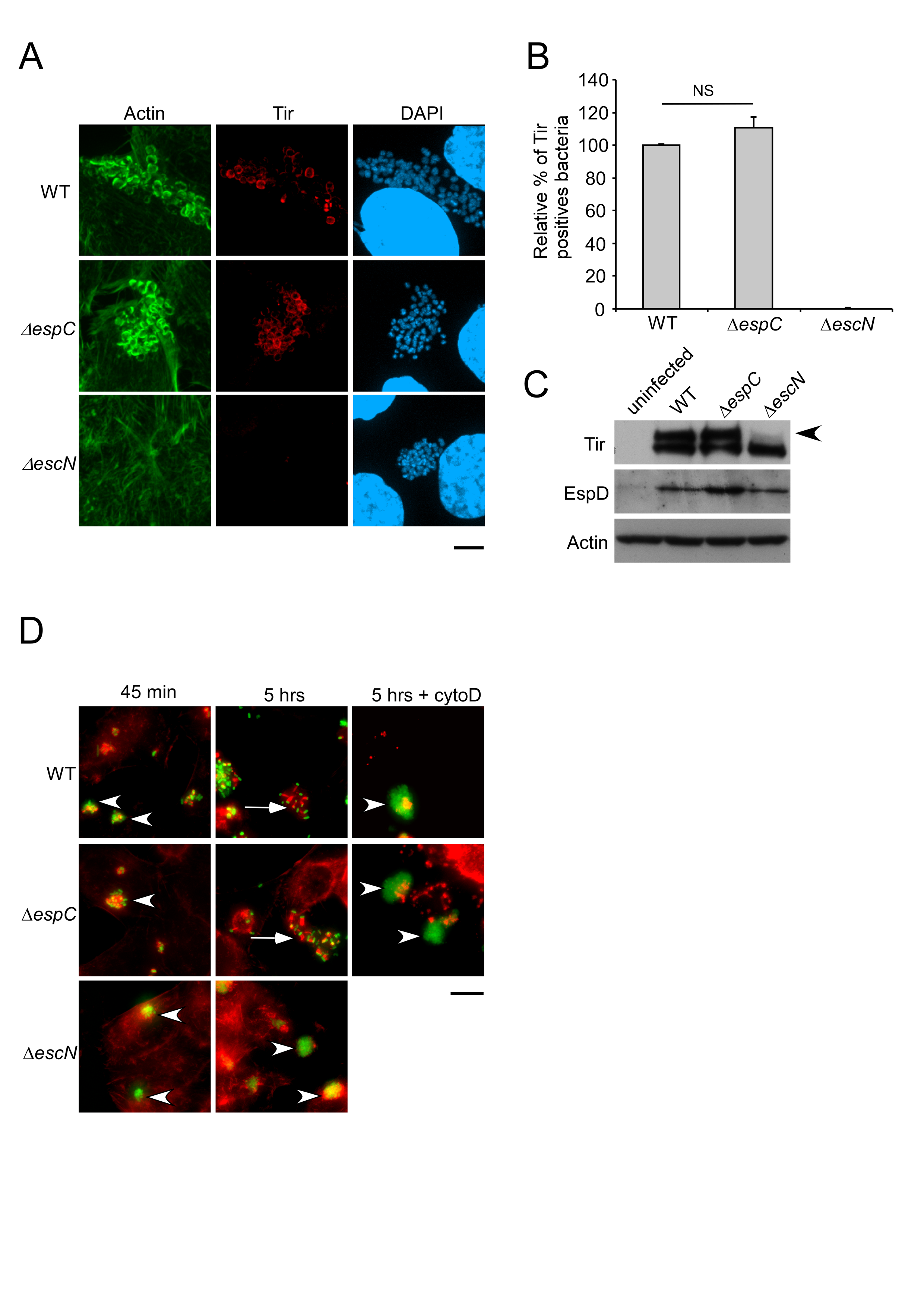

Supplement: S8 Fig — (A) HeLa cells were infected with primed EPEC strains for 45 min, fixed and processed for fluorescence staining of F-actin (green), Tir (red) and DNA (blue). Representative confocal micrographs of samples challenged with the strains indicated on the left. Scale bar: 5 μm. (B) The average percentage ± SEM of adherent bacteria showing Tir staining association. The values are representative of at least 500 adherent bacteria scored in 5 independent experiments. (C) HeLa cells were challenged with the bacterial strains, and whole cells lysates were subjected to Western Blot analysis using the indicated antibodies. Arrowhead: translocated Tir visualized as a larger migrating species [1]. No difference in Tir translocation could be detected between WT and ΔespC strains. These results indicate that EspC is dispensable for EPEC-induced actin reorganization and are consistent with previous observations that EspC does not affect signal transduction leading to A/E lesions [2]. (D) HeLa cells were infected with primed EPEC strains for 45 min or 5 hrs. Samples were fixed and processed for fluorescence staining of F-actin (red) and GFP-expressing-bacteria (green). Micrographs representative of at least 3 independent experiments are shown. Arrowheads indicate clusters of aggregated bacteria. Arrows show dispersed bacteria associated with actin pedestals. All strains formed tight bacterial clusters at 45 min. Bacterial dispersion was observed at 5 hrs p.i. for WT and espC mutant strain but not for the T3SS-deficient strain ΔescN. Bacterial dispersion was not observed when infection was carried out in the presence of cytochalasin D (cytoD). (TIF) [file ppat.1005013.s008.tif]

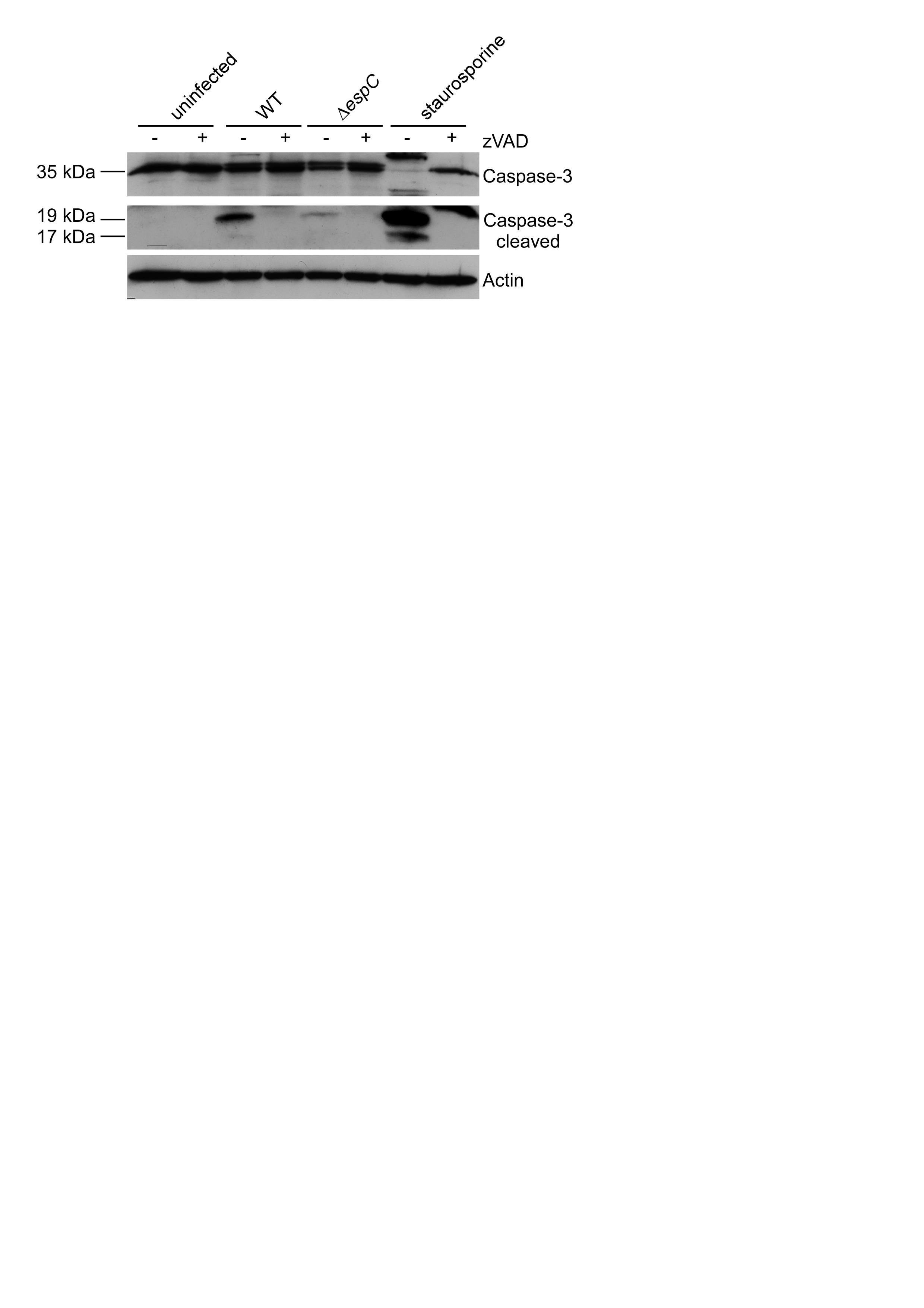

Supplement: S9 Fig — HeLa cells were treated with zVAD and challenged with primed EPEC strains for 45 min. Infection was carried on for 17 h in presence of gentamicin and zVAD. Samples were scraped in Laemmli sample buffer and subjected to Western Blot analysis using antibodies directed against full-length or cleaved caspase-3. Anti-actin Western-blot analysis is shown in the bottom panel as a loading control. Staurosporine was used as a positive control. Both WT and espC mutant strains induced caspase-3 cleavage, that was inhibited by zVAD. (TIF) [file ppat.1005013.s009.tif]
