# Supplementary material for: The Serine Protease EspC from Enteropathogenic Escherichia coli Regulates Pore Formation and Cytotoxicity Mediated by the Type III Secretion System
Source: PLoS Pathog. 2015 Jul 1;11(7):e1005013. doi: 10.1371/journal.ppat.1005013 (PMC4488501; doi:10.1371/journal.ppat.1005013)
Supplement: S1 Table — (PDF) [file ppat.1005013.s010.pdf]

**Strains**	**Characteristics**	Reference
WT E2348/69	EPEC wild-type strain	[3]
WT 85-170	EHEC wild-type strain	[4]
Δ*espC*	*espC* isogenic mutant of WT E2348/69 (*espC*::kan)	This study
Δ*espP*	*espP* isogenic mutant of WT 85-170 (*espP*::kan)	This study
Δ*espC* /pEspC^+	Δ*espC* strain complemented with pJLM174 grown in inducing conditions (arabinose)	This study
Δ*espC* /pEspC^-	Δ*espC* strain complemented with pJLM174 grown in repressing conditions (glucose)	This study
Δ*escN*	*escN* isogenic mutant of WT E2348/69 (*escN*::kan)	[5]
Δ*bfpA*	*bfpA* isogenic mutant of WT E2348/69 (*bfpA*::kan)	This study
Δ*espC* / Δ*bfpA*	*espC* and *bfpA* isogenic mutant of WT E2348/69 (*espC::kan ; bfpA*::cat)	This study
MAS111	*espC* mutant of WT E2348/69	[13]
**Plasmids**		
pJLM174	*espC* cloned into pBAD30	[6]
pJLM174-S256I	*espC*-S256I cloned into pBAD30	This study
pSat	*sat* cloned into pACYC184	[7]
pSepA	*sepA* cloned into pUC18	[8]
pEspP	*espP* cloned into Topo-blunt® vector	This study
