# Supplementary material for: The Serine Protease EspC from Enteropathogenic Escherichia coli Regulates Pore Formation and Cytotoxicity Mediated by the Type III Secretion System
Source: PLoS Pathog. 2015 Jul 1;11(7):e1005013. doi: 10.1371/journal.ppat.1005013 (PMC4488501; doi:10.1371/journal.ppat.1005013)
Supplement: S1 File — (PDF) [file ppat.1005013.s011.pdf]

Supporting information

The serine protease EspC from Enteropathogenic *Escherichia coli* regulates pore formation and cytotoxicity mediated by the Type III Secretion System

Julie Guignot, Audrey Segura and Guy Tran Van Nhieu

Bacterial culture and Priming

The bacterial strains and plasmids used in this study are listed in Table 1. All strains were grown in Luria-Bertani broth (LB) (17 hr at 37°C, 200 rpm). To activate the type III secretion system, EPEC strains were primed by diluting overnight LB culture (1:100 dilution) into DMEM containing 1g/L glucose and incubated at 37°C in a 5% CO_2 incubator for the indicating times [9]. When required, ampicillin was added to the medium at 100 μg/mL. When strains carrying pJLM174 were used, LB broth or DMEM were supplemented with 0.2% arabinose to induce or 0.2% glucose to repress EspC expression.

Antibodies and reagents

Rabbit polyclonal antibody directed against EspC was produced by Genescript Corporation (USA) using a KLH conjugate peptide (CNQVSSFEQPDWENR). Mouse monoclonal anti-EspB (clone B292) and anti-EspD (clone A84), kindly provided by F. Ebel, were described previously [10]. Rabbit polyclonal anti-Tir and anti-EspA have been described previously [11,12]. Anti-caspase 3 antibodies were from Cell signalling. Alexa-conjugated secondaries antibodies as well as Alexa-conjugated Phalloïdin were from Invitrogen (Life technology) and used at a dilution of 1:200 for immunofluorescence. The horseradish peroxidase-coupled antibodies were from Jackson Immunoresearch (West Grove, PA) and used at 1:20000 for Western blot analysis. Anti-actin directly coupled to horseradish peroxidase was from Cell Signaling and used at 1:20,000.

Phenylmethylsulfonyl fluoride (PMSF), Lucifer yellow CH dilithium salt (LY), the caspase inhibitor zVAD, and DAPI were from Sigma. The Complete ™ Protease inhibitor cocktail was from Roche diagnostic and used following the manufacturer's instructions.

Immunofluorescent staining

For staining of bacteria grown in DMEM or of eluted samples corresponding to the peak A and A/D of the MonoQ anion exchange chromatography, samples were incubated for 15 min

at 21°C with coverslips that were previously coated with poly-L-lysine at a final concentration of 50 μg / ml prior to fixation for 20 min in PBS containing 3.7 % paraformaldehyde (PFA). For cell challenge studies, following incubation with bacteria at indicated time, samples were washed with PBS and fixed with PFA. Following fixation, samples were blocked for at least 60 min at 21°C in PBS containing 10 % FCS. For infected cells, samples were permeabilized by incubation in PBS containing 0.1 % Triton-X100 for 4 min at 21°C. Samples were processed for immunofluorescent staining as previously described, using anti-EspA antibody at a dilution of 1:400, or anti-EspD antibody at a dilution of 1:300.

Cloning and generation of deletion mutants of *bfpA, espC,* and *espP* genes in EPEC and EHEC

The EHEC *espP* gene was amplified with its RBS sequence using the synthetic oligonucleotides 5'-GGA GCT TAT ATG AAT AAA ATA TAC TCT C-3' and 5'-TCA GAA CGA GTA ACG GAA ATT AGC G-3' and the PCR fragment was cloned into Topo-Blunt® vector (Invitrogen, Life technology). The construct was verified by DNA sequencing.

Mutations that delete the entire *espC* gene in EPEC or *espP* gene in EHEC or *bfpA* gene in EPEC were created by allelic exchange using the λ Red recombinase method. PCR fragments were generated using pKD4 as a template for *espC* and *espP* mutations and pKD3 for *bfpA* mutation, and a set of synthetic oligonucleotides, 5'-GGT TTC ATA ATG GAC TTC AGA TCT GGT GAT AAA AAC ATT ATG TGA AAA TCG TGT AGG CTG GAG CTG CTT C-3' and 5'- CGT GTC TAT AAA AAG GCC TGC CAG ATG GCA GGC CAG ATT TAA ACG TCT GCA TAT GAA TAT CCT CCT TAG-3' for *espC* ; 5'-CCT CTG ATA CAT TAG AAA AAC CAA TGT TTC CCT TAA AAA ATG GAG CTT ATG TGT AGG CTG GAG CTG CTT C-3' and 5'-GCA CTG AGC GTA AAG GGC CCG CAG GCC CTT TTG AAT ACG GAG TAC ATA TGA ATA TCC TCC TTA G-3' for *espP*, and 5'-CCG TGA CCT ATT AAT ACG GGG GTT TTA TAA GGA AAA CAG TTT TTG TGT AGG CTG GAG CTG CTT C-3' and 5'-CCT CCC ATA TAA TAC GCC CAA AAC AGG GCG TAT TAT GTA GAC ATA TGA ATA TCC TCC TTA G-3' for *bfpA*. PCR fragments carrying the kanamycin antibiotic resistance

aph(3')-II or chloramphenicol resistance gene flanked by regions homologous to the target locus were electroporated into WT EPEC E2348/69 strain or WT 85-170 EHEC strain carrying the λ Red recombinase expression plasmid pKD46. Isogenic mutant clones were verified by DNA sequencing. pKD46 was cured by growing mutant strains at 43°C.

Translocation assay

Cells were infected for 45 min at 37°C with primed bacteria. Monolayers were washed 3 times with ice-cold PBS containing 0.1 mM $CaCl_2$, 1 mM $MgCl_2$, and solubilized in 300 μL ice-cold PBS containing protease inhibitors and 0.1% Triton X-100 (TX100). All subsequent steps were performed on ice. Samples were scraped with a rubber policeman, transferred to a Dounce homogenizer, and subjected to 20 strokes. An aliquot of total cell lysates was analysed by Western blotting, while the rest were subjected to two successive centrifugations for 5 min at 20 000 g to remove cell debris and bacteria. Supernatants corresponding to the Triton-X100 soluble fraction containing the translocated proteins were analyzed by Western blotting using the highly sensitive detection reagent (SuperSignal West Femto Chemiluminescent Substrate, Thermo Scientific).

Proteinase K digestion

Insoluble forms of EspA, EspB and EspD released during overnight bacterial culture in DMEM were analyzed by proteinase K digestion. Bacterial pellets were incubated in DMEM containing 1 µg / mL proteinase K for 20 min at 20°C. Proteinase K was inactivated by addition of protease inhibitors (Complete^{TM}, Roche Pharmaceuticals) followed by immediate addition of Laemmli sample buffer and incubation for 10 min at 100 °C. Samples were subjected to Western blot analysis.

Caspase-3 activation

HeLa cells were infected with primed EPEC strains for 45 min at 37°C. The medium was removed and samples were further incubated for 1 additional hour in fresh medium.

Gentamicin was added to kill bacteria and samples were incubated for 17h. When mentioned, zVAD was added and maintained during all the duration of the experiment. Cells were scrapped in Laemmli sample buffer, transferred to an eppendorf tube and incubated for 10 min at 100 °C. Samples were subjected to Western blot analysis using antibodies as mentioned. Staurosporine was used at a final concentration of 1μM as a positive control for caspase-activation.

SUPPLEMENTAL REFERENCES

1. Warawa J, Kenny B (2001) Phosphoserine modification of the enteropathogenic Escherichia coli Tir molecule is required to trigger conformational changes in Tir and efficient pedestal elongation. Mol Microbiol 42: 1269-1280.

2. Stein M, Kenny B, Stein MA, Finlay BB (1996) Characterization of EspC, a 110-kilodalton protein secreted by enteropathogenic Escherichia coli which is homologous to members of the immunoglobulin A protease-like family of secreted proteins. J Bacteriol 178: 6546-6554.

3. Levine MM, Nataro JP, Karch H, Baldini MM, Kaper JB, et al. (1985) The diarrheal response of humans to some classic serotypes of enteropathogenic Escherichia coli is dependent on a plasmid encoding an enteroadhesiveness factor. J Infect Dis 152: 550-559.

4. Tzipori S, Karch H, Wachsmuth KI, Robins-Browne RM, O'Brien AD, et al. (1987) Role of a 60-megadalton plasmid and Shiga-like toxins in the pathogenesis of infection caused by enterohemorrhagic Escherichia coli O157:H7 in gnotobiotic piglets. Infect Immun 55: 3117-3125.

5. Garmendia J, Phillips AD, Carlier MF, Chong Y, Schuller S, et al. (2004) TccP is an enterohaemorrhagic Escherichia coli O157:H7 type III effector protein that couples Tir to the actin-cytoskeleton. Cell Microbiol 6: 1167-1183.

6. Mellies JL, Navarro-Garcia F, Okeke I, Frederickson J, Nataro JP, et al. (2001) espC pathogenicity island of enteropathogenic Escherichia coli encodes an enterotoxin. Infect Immun 69: 315-324.

7. Guignot J, Chaplais C, Coconnier-Polter MH, Servin AL (2007) The secreted autotransporter toxin, Sat, functions as a virulence factor in Afa/Dr diffusely adhering Escherichia coli by promoting lesions in tight junction of polarized epithelial cells. Cell Microbiol 9: 204-221.

8. Benjelloun-Touimi Z, Sansonetti PJ, Parsot C (1995) SepA, the major extracellular protein of Shigella flexneri: autonomous secretion and involvement in tissue invasion. Mol

Microbiol 17: 123-135.

9. Rosenshine I, Ruschkowski S, Finlay BB (1996) Expression of attaching/effacing activity by enteropathogenic Escherichia coli depends on growth phase, temperature, and protein synthesis upon contact with epithelial cells. Infect Immun 64: 966-973.

10. Ebel F, Deibel C, Kresse AU, Guzman CA, Chakraborty T (1996) Temperature- and medium-dependent secretion of proteins by Shiga toxin-producing Escherichia coli. Infect Immun 64: 4472-4479.

11. Hartland EL, Batchelor M, Delahay RM, Hale C, Matthews S, et al. (1999) Binding of intimin from enteropathogenic Escherichia coli to Tir and to host cells. Mol Microbiol 32: 151-158.

12. Knutton S, Rosenshine I, Pallen MJ, Nisan I, Neves BC, et al. (1998) A novel EspA-associated surface organelle of enteropathogenic Escherichia coli involved in protein translocation into epithelial cells. Embo J 17: 2166-2176.

13. Stein M, Kenny B, Stein MA, Finlay BB (1996) Characterization of EspC, a 110-kilodalton protein secreted by enteropathogenic Escherichia coli which is homologous to members of the immunoglobulin A protease-like family of secreted proteins. J Bacteriol 178: 6546-6554.
